# Atomistic simulations and network-based modeling of the Hsp90-Cdc37 chaperone binding with Cdk4 client protein: A mechanism of chaperoning kinase clients by exploiting weak spots of intrinsically dynamic kinase domains

**DOI:** 10.1371/journal.pone.0190267

**Published:** 2017-12-21

**Authors:** Josh Czemeres, Kurt Buse, Gennady M. Verkhivker

**Affiliations:** 1 Department of Computational and Data Sciences, Schmid College of Science and Technology, Chapman University, Orange, California, United States of America; 2 Department of Biomedical and Pharmaceutical Sciences, Chapman University School of Pharmacy, Irvine, California, United States of America; University of Minnesota Twin Cities, UNITED STATES

## Abstract

A fundamental role of the Hsp90 and Cdc37 chaperones in mediating conformational development and activation of diverse protein kinase clients is essential in signal transduction. There has been increasing evidence that the Hsp90-Cdc37 system executes its chaperoning duties by recognizing conformational instability of kinase clients and modulating their folding landscapes. The recent cryo-electron microscopy structure of the Hsp90-Cdc37-Cdk4 kinase complex has provided a framework for dissecting regulatory principles underlying differentiation and recruitment of protein kinase clients to the chaperone machinery. In this work, we have combined atomistic simulations with protein stability and network-based rigidity decomposition analyses to characterize dynamic factors underlying allosteric mechanism of the chaperone-kinase cycle and identify regulatory hotspots that control client recognition. Through comprehensive characterization of conformational dynamics and systematic identification of stabilization centers in the unbound and client- bound Hsp90 forms, we have simulated key stages of the allosteric mechanism, in which Hsp90 binding can induce instability and partial unfolding of Cdk4 client. Conformational landscapes of the Hsp90 and Cdk4 structures suggested that client binding can trigger coordinated dynamic changes and induce global rigidification of the Hsp90 inter-domain regions that is coupled with a concomitant increase in conformational flexibility of the kinase client. This process is allosteric in nature and can involve reciprocal dynamic exchanges that exert global effect on stability of the Hsp90 dimer, while promoting client instability. The network-based rigidity analysis and emulation of thermal unfolding of the Cdk4-cyclin D complex and Hsp90-Cdc37-Cdk4 complex revealed weak spots of kinase instability that are present in the native Cdk4 structure and are targeted by the chaperone during client recruitment. Our findings suggested that this mechanism may be exploited by the Hsp90-Cdc37 chaperone to recruit and protect intrinsically dynamic kinase clients from degradation. The results of this investigation are discussed and interpreted in the context of diverse experimental data, offering new insights into mechanisms of chaperone regulation and binding.

## Introduction

The 90 kDa heat-shock proteins Hsp90s belong to a class of highly abundant and evolutionary conserved molecular chaperones that are present in the cytosol of bacteria and in all eukaryotic organisms [**1–6**]. These chaperones manage late stages of conformational development, maturation and folding for a wide array of client proteins, including protein kinases and transcription factors that constitute a variety of signal transduction pathways and implicated in cell proliferation, differentiation, apoptosis and immune response [**7–18**]. The Hsp90 chaperone operates as a homodimer with a modular architecture in which each monomer consists of three distinct domains: an N-terminal domain (Hsp90-NTD) that harbors the ATP binding site and a mobile lid motif that mediates ATP-dependent dimerization of the NTDs, a middle domain (Hsp90-MD), which is implicated in binding of client proteins, and a C-terminal domain (Hsp90-CTD) that is required for constitutive dimerization [**12–18**]. Structural and biophysical studies have identified different conformational states of Hsp90 [**19–25**] that are associated with the main stages of the Hsp90-ATPase cycle. Hydrogen/deuterium exchange mass spectrometry (HX-MS) studies, electron microscopy (EM) and small-angle X-ray scattering (SAXS) experiments have characterized the thermodynamics and kinetics of structural transitions during the ATPase cycle, showing that these changes reflect stochastic fluctuations between preexisting conformational states, and are only weakly coupled to the nucleotide binding for the eukaryotic cytosolic Hsp90 [**26–32**]. In contrast to spontaneous conformational changes featured by eukaryotic Hsp90s, the ratchet mechanism of the ‘cochaperone-independent’ bacterial HtpG is strictly controlled by the nucleotide binding [**31**]. HX-MS studies of conformational dynamics for the HtpG, human Hsp90β, and yeast Hsp90 chaperones have unveiled that eukaryotic Hsp90s in their apo forms are considerably more flexible than the HtpG chaperones [**32**]. Functional assays of yeast Hsp90-NTD and Hsp90-MD mutants with reported effects on the ATPase activity were undertaken to show that the proper timing of conformational transitions is vital for client recruitment and processing by the chaperone [**33**]. According to this study, a client-specific residence time in the open conformational states is required for the normal cycle progression, and the excessive stabilization and prolonged occupation of the closed states could be detrimental for the client cycle. By incorporating specific fluorescence probes into yeast Hsp90 and employing single-molecule spectroscopy, kinetics of conformational changes in this chaperone has been recently dissected, revealing cooperativity of local and global motions during the ATPase cycle [**34**].

The integration of various cochaperones into the Hsp90 machinery can modulate progression of the Hsp90-ATPase cycle and ensure timely execution of protein client loading, activation, and release stages [**35–42**]. Importantly, cytosolic Hsp90 is the only form of the Hsp90 chaperones that are cochaperone-dependent for functional activities. The family of Hsp90 chaperones can be also regulated by post-translational modifications, which modulate dynamics and interactions with clients and cochaperones [**43–49**]. Cdc37 is a highly specialized cochaperone that in coordination with Hsp90 can facilitate conformational maturation, protein folding and acquisition of functional states for a large and diverse clientele of protein kinases [**50–54**]. The initial functional studies of protein kinase motifs involved in binding with the Hsp90-Cdc37 chaperone have revealed that multiple segments from the N-terminal and C-terminal kinase lobes cooperate in the client recruitment process [**55–59**]. A high-throughput study of the Hsp90-client interactions using luminescence-based mammalian interactome mapping has provided a first quantitative analysis of the Hsp90-client interactions, revealing that Cdc37 can distinguish kinase clients by recognizing their conformational instability, whereas cooperative Hsp90-Cdc37 binding must be invoked for thermodynamic sorting of strong and weak kinase clients [**60**]. HX-MS studies and functional assays of structurally similar client and nonclient kinases have confirmed this mechanism, demonstrating that kinase dependence on the Hsp90-Cdc37 chaperone is correlated with the degree of unfolding cooperativity, tryptophan accessibility and client compactness that favor exposure of the regulatory regions in the catalytic domain [**61**].

Using mutational analysis, this study showed that the interaction strength between Hsp90-Cdc37 chaperone and Src kinases can span a continuum spectrum of binding affinities from the weak client c-Src to the constitutive strong client v-Src kinase. These findings have suggested that similar molecular principles may underlie chaperone-kinase interactions for both weak and strong clients, and the propensity to expose proper interaction surfaces during recruitment to the chaperone may ultimately determine the interaction strength between a kinase and the Hsp90-Cdc37 system **[61**]. Recent studies have reaffirmed a central role of Cdc37 in chaperoning of kinase clients as silencing of Cdc37 function in cancer cells dramatically reduces binding of oncogenic kinase clients with Hsp90 [**62**]. By investigating the effects of Cdc37 mutants that disrupt the Hsp90-Cdc37 interactions on client binding, it was recognized that restricted Hsp90-Cdc37 interactions would not present an impediment for effective chaperoning of a wide range of kinase clients [**63**]. NMR spectroscopy mapping and fluorescence assays of the Hsp90-Cdc37 interactions with protein kinase clients have shown that these complexes could form cooperatively through an allosteric cross-talk between multiple interaction sites [**64**]. Molecular mechanisms underlying client recognition and thermodynamic sorting by the Hsp90-Cdc37 chaperone have been further explored by NMR approaches [**65**]. These illuminating experiments have established that Cdc37 can recognize kinase clients through a series of successive stages, in which the N-terminal domain of Cdc37 (Cdc37-NTD) may perform primary screening of kinase clients and induce partial disorder and the enhanced heterogeneity of kinase ensembles, while the C-terminal domain regions (Cdc37-M/C) would provide additional protection of partially unfolded client states. Accordingly, Cdc37 may expand its ‘duties’ as a chaperone adaptor and serve a primary ‘sensor’ of kinase instability that triggers a cascade of allosteric changes that promotes local unfolding and effective sorting of client substrates with the aid of Hsp90 [**65**].

**Fig 1:**
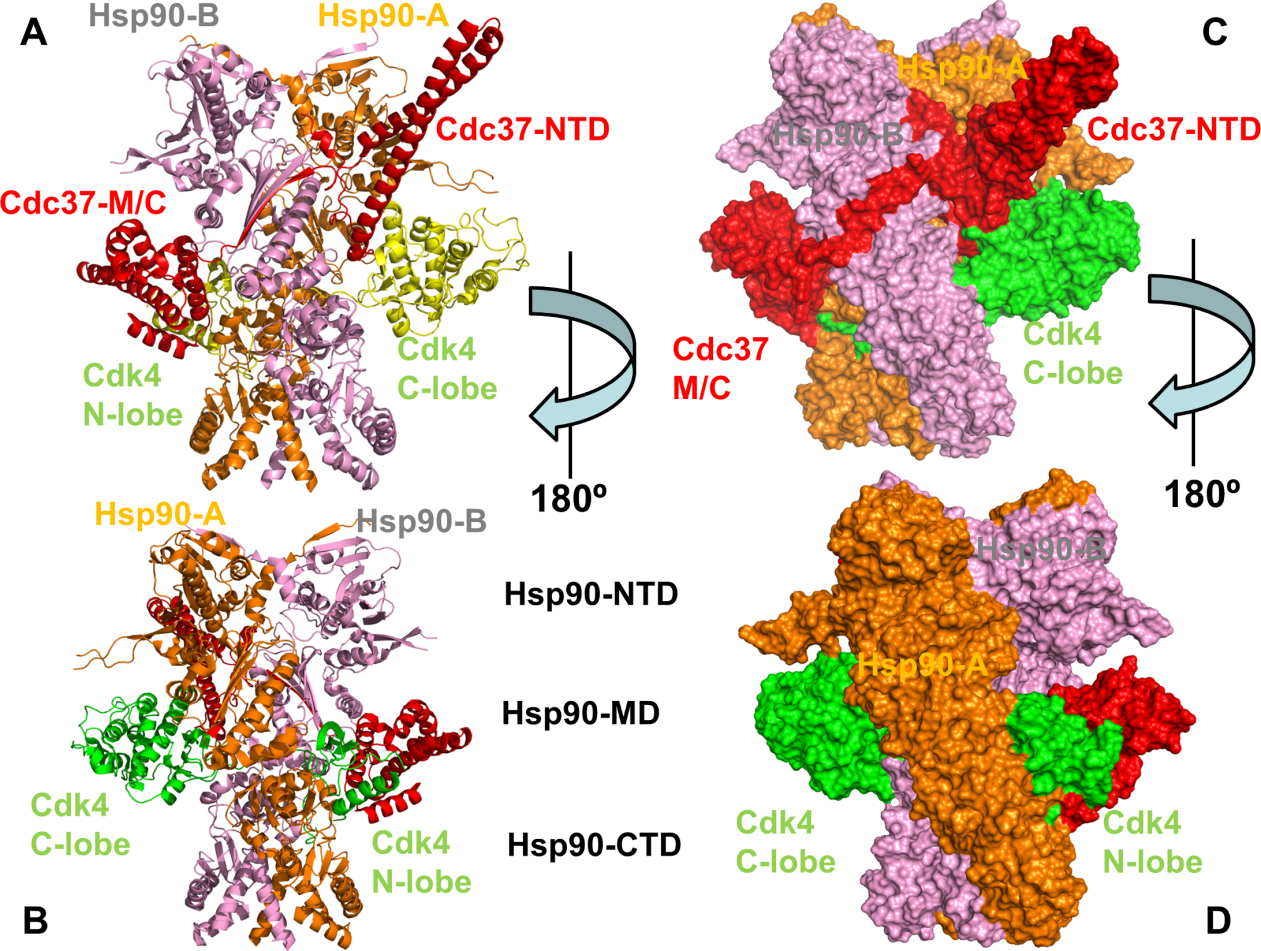
Structural organization of the Hsp90-Cdc37-Cdk4 assembly. The intertwined structure of the human Hsp90-Cdc37-Cdk4 complex is shown in ribbons (A, B) and surface representation (C, D). Two 180º views of the structure are presented. The Hsp90-A monomer of the dimer is shown in orange, Hsp90-B is shown in pink, Cdc37 is depicted in red, and Cdk4 client is shown in yellow. The individual domains of Hsp90, Cdc37 and Cdk4 are annotated. Each monomer in the full length human Hsp90β protein is divided into three domains. In the Hsp90-A monomer, the domains are annotated as follows: NTD (residues 1–215), MD (residues 216–552), and CTD (residues 553–690). In the Hsp90-B monomer, NTD (residues 1–215), MD (residues 216–552) and CTD (residues 553–691). The domain annotation is consistent with sequence analysis of Hsp90 proteins [**71, 72**] and functional studies of Hsp90 constructs [**73–75**]. In the structure, the functionally relevant charged linker region [**76–78**] corresponds to residues 222–295 with a fragment of the linker (residues 221–273) not resolved in the original structure. Cdc37 domains are annotated as follows: Cdc37-NTD (residues 1–147), Cdc37-M/C (residues 148–260). The Cdk4 N-lobe (residues 1–99) and Cdk4 C-lobe (residues 100–295) annotations are consistent with the original study [**69**].

Structural and thermodynamic characterization of the Hsp90-Cdc37 binding with protein kinase clients have been constrained by difficulties associated with the dynamic changes in client and chaperone conformations and a transient character of chaperone-client interactions. Multiple flexible regions in the Hsp90 and Cdc37 chaperones were implicated as potential binding sites for client interactions, suggesting that the intermolecular binding interfaces undergo structural and dynamic remodeling during client binding and release cycle [**66, 67**]. The initial EM reconstruction of the Hsp90-Cdc37-Cdk4 kinase complex unveiled an asymmetric assembly and a broad network of interactions formed by the Hsp90 dimer with Cdc37 cochaperone and Cdk4 [**68**]. The EM-based analysis of the Hsp90-Cdc37-Cdk4 complex offered insight into the stoichiometry, topology and shape of this multi-protein assembly, but the atomistic details of the binding interactions remained hidden. The cryo-EM structure of the human Hsp90-Cdc37-Cdk4 kinase complex [**69**] marked a fundamental breakthrough in the field, revealing a complex and highly intertwined arrangement in which the Hsp90 dimer and Cdc37 trap a partially disordered kinase state (**Fig 1**). In this structure, Hsp90 dimer assumes a symmetrical closed conformation, which closely resembles an ATP-bound closed state of yeast Hsp90 [**19**]. A partially unfolded N-lobe is implanted inside the Hsp90 dimer and stabilized by the interactions with Hsp90-MD and Hsp90-CTD (**Fig 1**). A number of unique features and several major intermolecular interfaces that characterize the underlying topology of the Hsp90-Cdc37-Cdk4 complex indicated that allosteric interactions between binding sites are delicately tuned to enable client recognition and progression of the chaperone cycle. Based on structural characterization of the Hsp90-client interactions, Agard and colleagues have proposed a model of the chaperone cycle [**69**] according to which Cdc37 initially sequesters an open kinase conformation and the resulting intermediate binds to a client-loading open form of the Hsp90 dimer. During the functional cycle, the Hsp90 chaperone progresses from a relaxed form to a closed ATP-bound state that effectively traps a partially unfolded kinase client, leading to the formation of the Hsp90-Cdc37-Cdk4 complex. Upon ATP hydrolysis, Hsp90 opens up and releases the folded kinase client from the chaperone system. Based on their pioneering work, Agard and colleagues have also inferred that dynamic Hsp90-client interactions may have evolved to enable rapid and diverse kinase responses in signaling networks, where the chaperone role is to protect unstable kinases from degradation and suppress an undesirable basal activity [**70**]. Importantly, this study has indicated that even mature kinases can maintain chaperone dependence, as Hsp90-addicted kinase clients could frequently sample a partially unfolded state even after folding. Collectively, the body of structural and functional investigations has provided evidence that Cdc37 can serve as a recognition scaffold for unstable kinase clients, but requires cooperation with Hsp90 to properly execute and complete cycle of client maturation, folding and release. However, the molecular details of this highly dynamic binding mechanism that operates through allosteric coordination of several spatially separated binding interfaces remain largely elusive. In addition, the structure of the chaperone-kinase complex provided only a single snapshot of a highly dynamic assembly formed through cooperative allosteric interactions. Current understanding of allosteric communication and long-range signaling that underlie the Hsp90-Cdc37 interactions with client kinases remains rather simplistic and incomplete to explain diverse experimental data on mutations affecting allosteric regulation and client binding.

Computational studies have also elucidated molecular details of Hsp90 dynamics and binding with cochaperones and inhibitors to quantify mechanisms of allosteric regulation in Hsp90. Our initial studies have provided evidence for allosteric communications in Hsp90 by showing that coordinated chaperone movements enable efficient signal transmission between the NTD and CTD binding sites [**79–81**]. Using these early insights, we combined computational and experimental approaches in structural characterization of the C-terminal binding site of Hsp90 and discovery of allosteric inhibitors [**82–84**]. Atomistic molecular dynamics (MD) simulations and coarse-grained approaches have systematically examined conformational dynamics and collective motions in different Hsp90 states [**85, 86**]. By combining MD simulations and structure-based network analysis, we have characterized conformational dynamics and allosteric interactions in the Hsp90 complexes with various cochaperones and client proteins [**87–90**]. These studies have shown that the residue interaction networks in the Hsp90 complexes with p53 [**89**] and client recruiter cochaperones Cdc37, Sgt1 and Rar1 [**90**] form small-world topologies in which key mediating centers of the global communications determine functional hotspots of chaperone regulation. The network-centric modeling of the Hsp90 interactions has suggested that allosteric coupling between key functional centers is required in regulation of the inter-domain communications, control of ATP hydrolysis, and protein client binding [**88–90**]. Computational design and functional assay experiments of mutations that control allosteric points of Hsp90 have characterized structural and dynamic signatures of allosteric Hsp90 switches [**91**]. A cross-talk between allosteric ligand binding and dynamic changes in the Hsp90 has been explored to characterize mechanisms of allosteric activation in the chaperone [**92**]. Collectively, computational studies indicated that the interactions of Hsp90 with cochaperones and client proteins are highly dynamic and can elicit a cascade of conformational changes through modulation of structural stability and flexibility in the interacting proteins.

In the current work, we performed all-atom molecular dynamics (MD) simulations of the Hsp90 and Cdk4 structures in different functional states. Multiple independent MD simulations were carried out for the Cdk4-cyclin D1/D3 complexes, a monomeric unbound form of Cdk4, a client-free Hsp90 dimer and the Hsp90-Cdc37-Cdk4 complex. Atomistic simulations and distance fluctuation analysis of MD trajectories were combined with the energy analysis of binding interfaces to examine stability of different functional states and identify regulatory hotspots that may control client recognition. We present evidence supporting an allosteric mechanism in which client binding can trigger coordinated dynamic changes and induce global rigidification of the Hsp90 inter-domain regions that is coupled with a concomitant increase in conformational flexibility of the kinase client. By combining conformational dynamics with the rigidity decomposition analysis and network-based emulation of thermal unfolding, our study suggested that Hsp90-Cdc37 chaperone may exploit differential stabilization of the kinase lobes and target weak spots of highly dynamic Cdk4 client to induce and protect a non-native, partially unfolded kinase state. The results of this investigation are discussed and interpreted in the context of diverse experimental data, offering new insights into mechanisms of chaperone regulation and binding.

## Results and discussion

### Atomistic simulations of the Hsp90 chaperone in different functional forms: Client-induced Modulation of conformational ensembles couples local and global dynamic changes

Using an ensemble-based model of allosteric regulation [**93–97**], we examined several stages of the chaperone-client cycle by simulating conformational dynamics of the Hsp90 chaperone and Cdk4 structures in their different unbound and client-bound functional states. The central hypothesis of this analysis assumes a mechanism of client recognition by the chaperone system in which modulation of structural and dynamic changes in the interacting proteins controls progression of the chaperone and kinase life cycles. The cryo-EM structure of the human Hsp90-Cdc37-Cdk4 kinase complex [**69**] showed a complex and intertwined arrangement of the interacting modules (**Fig 1**). In this structure, Hsp90 dimer assumes a closed conformation and the open structure of the kinase is in a partially unfolded state, with its two lobes on separate sides of Hsp90 and with the intervening region in an elongated nonnative conformation. The partially unfolded N-lobe region of the kinase client is threaded through the cleft between the Hsp90 monomers and stabilized by hydrophobic contacts with the client-binding site that mimic the native interactions between the kinase lobes. The crystal structures of phosphorylated Cdk4 bound to cyclin D revealed a dynamic Cdk/Src-like inactive kinase conformation in these complexes, showing that cyclin binding and phosphorylation are still not sufficient to trigger activation conformational transitions. Importantly, the activation mechanism of Cdk4 client is distinct from nonclient members of Cdk family [**60**] and requires synchronization of multiple events including cyclin binding, phosphorylation and substrate binding to achieve full activation [**98,99**]. It remains unclear why chaperone-induced trapping of an open Cdk4 conformation can benefit its activation. As a possible explanation, it was suggested that a lobe-separated, open Cdk4 conformation could create favorable thermodynamic and kinetic conditions for efficient ATP binding, which in turn would stabilize the kinase active state and trigger subsequent release of the folded kinase from chaperone system [**100–102**]. It was also argued that the increased dynamics of the open kinase state can be beneficial for rapid access and binding of substrates that are essential for Cdk4 activation [**102**].

**Fig 2:**
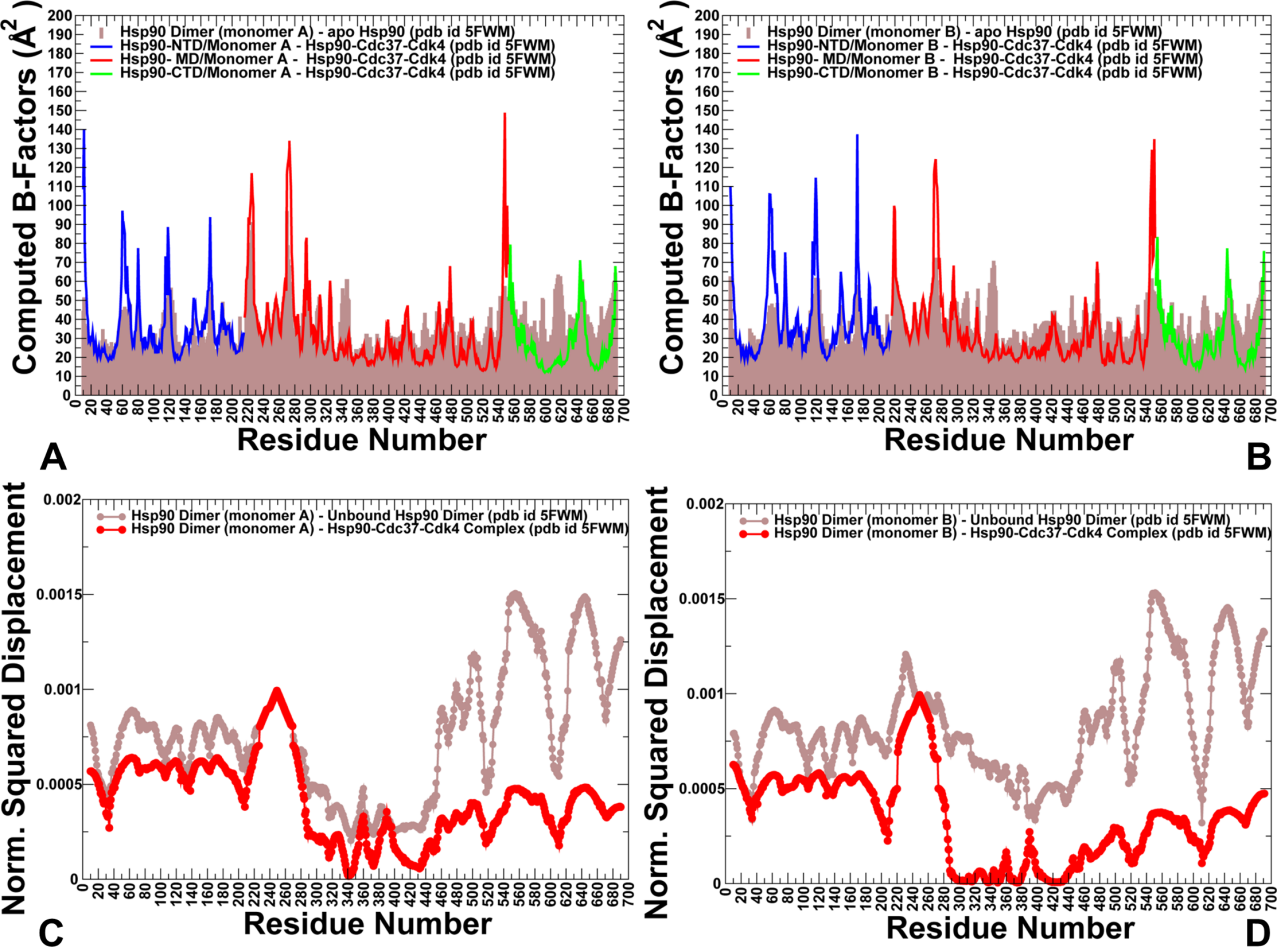
Conformational dynamics and PCA modes in the unbound and client-bound Hsp90 dimers. Conformational dynamics profiles of the Hsp90-monomer (A) and Hsp90-B monomer (B) in the unbound forms (shown in brown filled bars) and in the complex (shown in colored lines). Residue-based conformational mobility profiles in the complex are shown as lines with filled squares and colored as follows: Hsp90-NTD (residues 1–215) in blue, Hsp90-MD (residues 216–552) in red, and Hsp90-CTD (residues 553–690) in green. (C, D) The normalized squared displacement of the Hsp90 residues averaged over first three PCA components are shown for the unbound (in brown lines) and bound Hsp90 dimer (in red lines). The slow mode profile for Hsp90- monomer a (panel C) and for Hsp90-monomner B (panel D).

To address these questions, we simulated conformational dynamics of the Hsp90 chaperone and Cdk4 structures in their native and client-bound functional states. We tested a hypothesis that Cdk4 catalytic domain may be predisposed for recruitment to the chaperone system by featuring an intrinsically dynamic structure with the excessively flexible N-lobe regions–a common attribute of kinase clients that may be exploited by the chaperone to induce partial unfolding of kinase client upon recruitment to the Hsp90-Cdc37-Cdk4 complex. First, we compared conformational dynamics of the unbound Hsp90 dimer and client-bound Hsp90 in the Hsp90-Cdc37-Cdk4 complex. In this analysis, we examined whether binding could alter conformational dynamics of the Hsp90 dimer and cause local or global redistribution of conformational mobility in functional regions of the binding partners. Conformational mobility of protein residues was evaluated by using the mean square residue fluctuations and computed B-factors. We specifically focused on changes in the conformational variations of the Hsp90 regulatory motifs, the inter-domain and intermolecular interfaces (**Fig 2**). The increased stabilization of the Hsp90 dimer in the chaperone-client complex propagated from the interfacial regions to the Hsp90-MD and Hsp90-CTD regions (**Fig 2A and 2B**). The Hsp90-MD residues in the core of the complex and the three-helix bundle (residues 398–453) exhibited the reduced thermal fluctuations, as these regions were stabilized by the intra- and intermolecular interactions. Consistent with the HX-MS experiments [**32**], conformational dynamics showed the increased stability of the Hsp90-MD clusters 420–429, 472–484 and the MD-CTD interfacial region (residues 513–519) (**Fig 2A and 2B**). The intermolecular binding interfaces displayed reduced fluctuations, including the Hsp90-MD segment (residues K399, K402, R405, and K406) involved in interactions with Cdc37-NTD and Hsp90 residues F341, L343, F344, L611 and M602 that interact with the disordered kinase region (**Fig 2A and 2B**). Of special significance were the markedly reduced conformational fluctuations and stabilization of the recognition Src loop (residues 341-FDLFENKKKK-350), and the Hsp90-CTD helix 15 (residues 599–611) that displayed highly mobility in the unbound Hsp90 dimer [**32**]. The hydrophobic motif of the amphipathic Src-loop maintained stabilizing contacts with the Cdc37-M/C domain and kinase N-lobe during simulations and remained largely immobilized in the complex. A noticeable redistribution of conformational dynamics was also observed in the Hsp90-CTD helix 15, where the extensive intra- and intermolecular contacts formed by M602, L611, and R612 residues promoted stabilization of the Hsp90-CTD regions (**Fig 2A and 2B**). Our findings revealed that conformational dynamics of the Hsp90 near the Hsp90-CTD dimerization regions and binding interfaces with Cdk4 was considerably suppressed. Importantly, however, client binding induced structural stabilization that propagated beyond the binding interface residues. The integration of the Cdk4 client to the Hsp90-Cdc37 system incurred global changes in the dynamics of the Hsp90 dimer by narrowing the accessible conformational space and reducing flexibility of the MD and CTD regions in both monomers. The quenching of local dynamics in the MD and CTD domains was balanced by the increased flexibility of the Hsp90-NTD regions and strengthening of the inter-domain couplings (**Fig 2A and 2B**). These coordinated dynamic changes can be functionally significant to facilitate client binding and ensure proper dwell time of the client-bound conformational state during the chaperone cycle. Overall, local quenching of subdomain dynamics in the MD and CTD regions of Hsp90 can be counterbalanced by strengthening of the inter-domain correlations and inter-molecular couplings. A similar pattern of dynamic changes induced by allosteric activators was detected in other protein systems that are primarily regulated by dynamically-driven allostery [**103–105**].

To characterize functional motions and collective dynamics of the Hsp90 chaperone, we used Principal Component Analysis (PCA) [**106,107**] of MD trajectories and compared the slow mode profiles averaged over first three PCA components for the unbound and bound forms of Hsp90 (**Fig 2C and 2D**). The maxima along the slow mode profiles correspond to regions undergoing global structural movements, while the local minima describe immobilized hinge positions that coordinate collective changes. The slow mode shapes for the unbound and client-bound forms of the Hsp90 dimer revealed a considerable similarity, supporting the notion that conservation of major hinge centers and preexisting pathways of functional changes are determined by the native protein topology. In the unbound form, the slow mode profiles revealed several minima in the Hsp90-MD corresponding to hydrophobic residues P287, W289, F341, L343, Y356, F376, L369, I370, I400, and L401 (**Fig 2C and 2D**). Structural mapping of the Hsp90 hinge clusters pointed to their strategic location near the inter-domain regions (**S1 Fig**). In particular, the inter-domain coupling in the Hsp90 dimer are determined by the stable hinge cluster formed at the NTD-MD interface by F208 from the NTD and MD residues P287, W289, Y356 and V360. These observations are consistent with the experimentally observed critical role of this hydrophobic cluster to stabilization of the NTD-MD interface in the Hsp90 chaperones [**108**].

**Fig 3:**
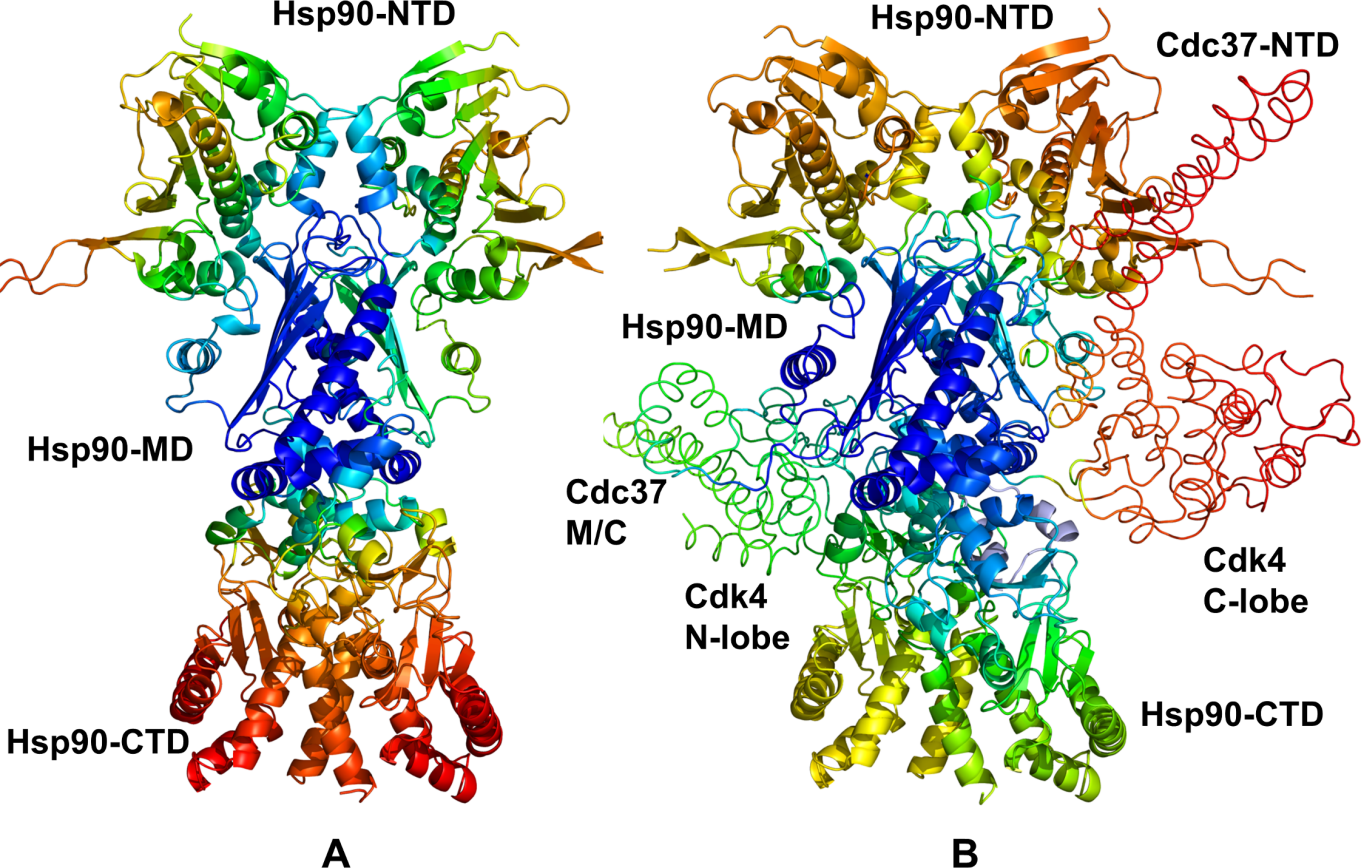
Structural mapping of functional dynamics in the unbound and bound Hsp90 forms. Structural mapping of the of the conformational mobility profiles for the unbound Hsp90 dimer (A) and Hsp90-Cdc37-Cdk4 complex (B). Conformational dynamics profiles were computed by averaging protein motions in the space of five lowest frequency modes. The color gradient from blue to red indicates the decreasing structural rigidity (or increasing conformational mobility) of the protein residues. The Cdc37-NTD, Cdc37-M/C domain and Cdk4 kinase lobes are annotated and their positions are indicated.

Structural maps of collective dynamics driven by the slowest PCA modes in the unbound (**Fig 3A**) and client-bound Hsp90 dimers (**Fig 3B**) highlighted client-induced partial redistribution and migration of the hinge centers in the complex. We observed relocation of major hinge clusters from the NTD and NTD-MD regions in the unbound Hsp90 dimer to the MD-CTD regions in the Hsp90-Cdc37-CdK4 complex (**Fig 3**). In the Hsp90-Cdce37-Cdk4 complex, the hinge centers in the Hsp90-MD and MD-CTD inter-domain regions (F341, L343, Y356, I370, and F376) expanded and formed interacting local clusters that further strengthened stability of these coordinating sites. Several hinge residues in the Hsp90-MD were organized in the cluster L369-I370-V381-L401-I404 located at the binding interface with Cdk4-NTD (**S1 Fig**). In the MD-CTD inter-domain region, hinge residues W598, M602, M606, L611, and R612 were colocalized and formed a hinge center that mediated the intermolecular coupling with the client kinase (**S1 Fig**). Importantly, the mobility of the recognition Src-loop (residues 341-FDLEF-345) and MD-CTD interfacial regions (residues 598–612) was markedly reduced in the slow modes, giving rise to the formation of a major hinge center at the interface with the client kinase.

### Exploring conformational dynamics of the Cdk4 client: Differential stabilization of kinase lobes and highly flexible N-lobe are #ntrinsic characteristics of dynamic kinase clients

**Fig 4:**
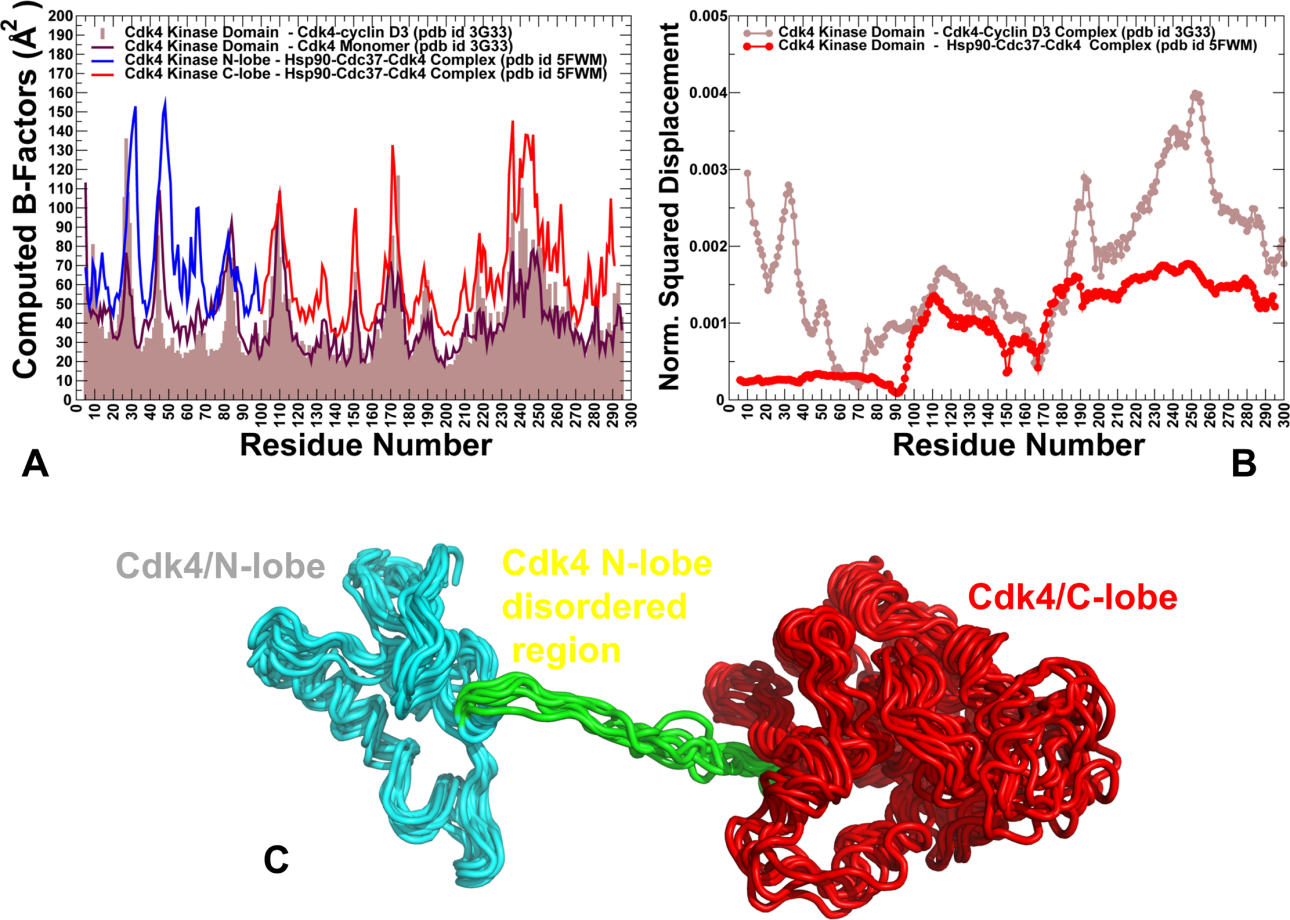
Conformational dynamics of Cdk4 in different functional states. (A) Conformational dynamics profile of Cdk4 catalytic domain in the complex with cyclin D3 (pdb id 3G33) is shown in brown bars and serves a reference for comparison with mobility profiles in other functional states. Conformational dynamics profile of the unbound, monomeric Cdk4 form is shown in maroon lines. Cdk4 mobility in the Hsp90-Cdc37-Cdk4 complex is shown for N-lobe in blue lines) and C-lobe in red lines. (B) The normalized squared displacement of the Cdk4 domain residues averaged over first three PCA components are shown for the Cdk4-cyclin D3 complex (in brown lines) and for the Hsp90-Cdc37-Cdk4 complex (in red lines). (C) An overlay of representative Cdk4 conformations from the MD ensemble of the partially disordered Cdk4 client in the Hsp90-Cdc37-Cdk4 complex. The ordered portion of the N-lobe is shown in cyan ribbons, a disordered region of the N-lobe is in green ribbons, and the ordered C-lobe is in red ribbons. Notice a significant conformational mobility and inflated nature of the ordered regions, while disordered region is shielded by the Hsp90 interactions.

To characterize structural and dynamic changes in the Cdk4 client, we explored conformational landscape of the native Cdk4 domain by simulating crystal structures of Cdk4/cyclin D1 [**98**] and Cdk4/cyclin D3 [**99**] complexes. MD simulations of the Cdk4-cyclin D3 crystal structure revealed a considerable conformational flexibility of the inactive Cdk4 conformation that was especially evident in the N-lobe regions, including the αC-helix (residues 50–67), αC-β4 loop (residues 67–74) and β4-β5 sheet (residues 75–95) (**Fig 4A**). Due to the increased flexibility of the N-lobe regions proximal to the αC-helix/αC-β4-loop, we observed local movements of the αC-helix, often deviating from the fully inactive ‘out’ position. These results are consistent with the HX-MS studies that suggested that the increased exposure and greater backbone hydrogen exchange near activation regions is a unifying characteristic of kinase clients [**61**]. In the Cdk4-cyclin D3 complex (pdb id 3G33), the Gly-rich β3-αC loop experienced considerable thermal fluctuations (**Fig 4A**). The elevated mobility in this region could remove steric constraints on positional preferences of the adjacent αC-helix which explains conformational variations of the αC-helix between the inactive and active-like conformations. MD simulations also showed that the C-lobe core became less compact as the central αE-helix (residues 113–134) and αF-helix (residues 193–210) displayed appreciable thermal fluctuations. Significant dynamic changes were also detected an inter-lobe regions, including the αD-helix (residues 99–107) and a small proline-rich loop (108-PPPGLP-113) connecting the αD-helix and αE-helix. These findings indicated that the intrinsic dynamics of Cdk4 can result in the reduced allosteric coupling between functional regions and lower rigidity of the kinase fold. These factors can determine client status of Cdk4 and create a favorable dynamic environment for chaperone intervention. Hence, Cdk4 can be predisposed for recruitment to the chaperone system by featuring an intrinsically dynamic catalytic domain.

We also analyzed conformational dynamics of Cdk4 in the Hsp90-Cdc37-Cdk4 complex. The client kinase is held by the Hsp90-Cdc37 in a spatially confined, yet highly dynamic ensemble. The results revealed appreciable fluctuations of Cdk4, particularly in partially unfolded N-lobe regions β4 (residues 75–85), β5 (residues 86–93) and αC-β3 loop (46-NGGGGGGGL-54) (**Fig 4A**). A comparison of slow modes profiles for the Cdk4 client revealed conservation of hinge residues located near the inter-lobe region that is anchored by Cdc37-NTD (**Fig 4B)**. Overall, the ensemble of Cdk4 conformations displayed signatures of a swollen globule-like state [**109**], where the folded C-lobe and ordered N-lobe region of Cdk4 client were mobile showing and inflated structure with packing interactions broken. At the same time, the unfolded portion of the N-lobe is shielded and partially stabilized by the Hsp90 interactions (**Fig 4C**).

To quantify the extent of protection of the kinase residues in the complex, we also evaluated the residue depth (RD) values by measuring the average residue exposure in the MD ensemble [**110–112**]. The results showed that the hydrophobic regions near the αC-helix (residues 50–67), αC-β4 loop (residues 67–74) and a β4-β5 sheet (residues 75–95) are indeed protected in the complex (**S2 Fig**). Hence, integration of Cdk4 client into the Hsp90 system results in spatial confinement of the dynamic Cdk4 conformational ensemble that is shielded by stabilizing contacts of the Hsp90 binding region. This can facilitate the release of water molecules from the interacting proteins and favor dynamic ensemble that is reminiscent of so-called dry molten globule intermediate that can occur at the initial stage of unfolding [**109**]. A similar compaction of conformational ensembles in client proteins upon recruitment to the chaperone was observed in the NMR studies of chaperone Spy and its soluble client protein Im7 [**113**], and for clients bound to the periplasmic trimeric chaperone Skp [**114,115**]. Noteworthy, Hsp90 provides a significantly larger surface area for interactions with substrate proteins than other chaperones [**116,117**]. It was proposed that client recognition by the Hsp90 can be based on distributing weak hydrophobic interactions over a fairly large surface that is supported by some electrostatic interactions. Intriguingly, our findings that a flexible Src-loop in the Hsp90-MD plays an important role in the dynamic exchange with the client kinase corroborated with several structural studies suggesting that this chaperone region is involved in binding with many partially disordered protein clients. In particular, NMR-based structural analysis of the Hsp90 interactions with the intrinsically disordered Tau protein revealed the binding site near the amphipathic Src-loop of Hsp90, suggesting that similar binding principles can be exploited by the chaperone in recruitment of distinct client proteins [**118**].

In general, by comparing conformational dynamics of the chaperone and client kinase in different functional states, we found that global propagation of rigidity in the Hsp90 binding regions can be coupled with the instability of the kinase client. A mechanism of reversible entropy transfer where the ordering of the chaperone flexible regions is accompanied by reciprocal unfolding or increased dynamics of the bound substrate was previously recognized as a distinct mode of chaperone function that is particularly well-suited for the chaperone cycles of repeated client recruitment and release [**119,120]**. Our results indicated that a similar mechanism may be also operational during client recognition and integration into the Hsp90-Cdc37 chaperone complex.

**Fig 5:**
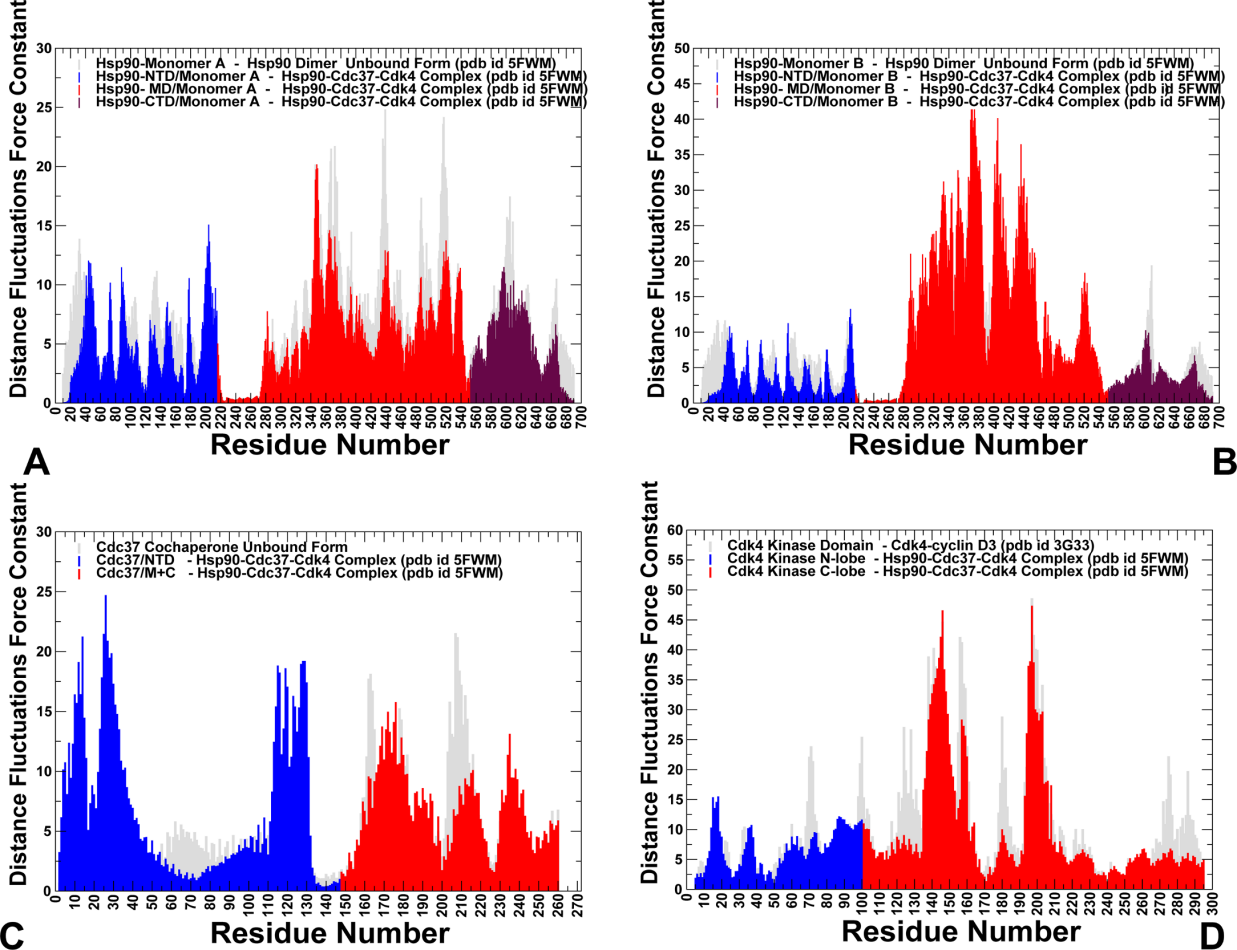
The distance fluctuations analysis and residue-based force constant profiles of the unbound and client-bound Hsp90 forms. The distance fluctuation force constant profiles of the Hsp90-monomer (A), Hsp90-B monomer (B) and Cdk4 kinase client (C) in the unbound forms (shown in grey light bars) and in the complex (shown in domain-colored bars). Residue-based force constant profiles in the complex are shown in bars and colored as follows: Hsp90-NTD (residues 1–215) in blue, Hsp90-MD (residues 216–552) in red, and Hsp90-CTD (residues 553–690) in maroon. (C) The Cdc37-NTD (residues 1–147) is shown in blue, Cdc37-M/C (residues 148–260) in red. (D) Cdk4-N lobe (residues 1–99) is shown in blue, and Cdk4 C-lobe (residues 100–295) is in red.

Distance fluctuations analysis of the conformational ensembles was used to probe structural stability and allosteric communication propensities of the Hsp90 and Cdk4 residues in the unbound and client-bound chaperone forms. We computed the residue-based distance fluctuation force constants that measure the energy cost of the residue deformation during simulations [**121–123**]. In this approach, structural rigidity of functionally important regions is associated with their high stability indexes and small fluctuations in their average inter-residue distances. Our previous studies showed that distance fluctuations profiles and respective force constant indexes can be related with allosteric residue propensities, as the mean square fluctuations between a pair of residues provides an efficient measure of the signal commute time [**87–90**]. According to this model, stable residues that display small fluctuations in their distances to other residues correspond to effectively communicating rigid sites and can serve as allosteric hotspots (or ‘signal dispatchers’). We first compared the distance fluctuation force constant profiles in the unbound and bound forms of the Hsp90 dimer (**Fig 5A and 5B**). This analysis revealed client-induced differential changes in the stability of the Hsp90 monomers. While the stability of the Hsp90-MD residues in the first monomer was moderately decreased (**Fig 5A**), a significant stabilization of the Hsp90-MD regions was seen in the second monomer B (**Fig 5B**), reflecting strengthening of both the inter-domain interactions and intermolecular contacts with Cdc37 and Cdk4 binding partners. Notably, functional hinge centers corresponded to the sharp peaks in the force constant distribution. Of particular interest was the increased distance fluctuation force constants and corresponding rigidification of the recognition Src-loop residues 341-FDLF-344 that are involved in the intermolecular contacts with the disordered kinase regions. More importantly, the predicted allosteric centers in the Hsp90-MD (L369, I370, V381, L401, and I404) and Hsp90-CTD regions (W598, M602, M606, L611, R612) formed large stable clusters that rigidified structural environment beyond the local binding interfaces.

A global expansion of stable allosteric clusters induced by client binding was observed in the Hsp90-MD and Hsp90-CTD regions that also solidified the MD-CTD inter-domain couplings (**Fig 5A and 5B**). In addition, we registered large force constant values for the Cdc37-NTD residues that are involved in binding with the Hsp90-MD and anchor Cdk4 C-lobe **(Fig 5C**). On the other hand, the force constant values for Cdk4 N-lobe residues were reduced as compared to the native Cdk4 structure (**Fig 5D**). The observed redistribution of allosteric hotspots supported our findings that major centers that control signal transmission in the complex migrated to the Hsp90-MD/CTD regions and Cdc37-NTD interfacial residues. Moreover, a global rigidification and the enhanced mediating capabilities of these Hsp90 and Cdc37 regions can be contrasted with the increased instability of Cdk4 residues. These results supported a model of reciprocal dynamic changes between Hsp90 and Cdk4 in which client binding can induce expansion of allosteric centers in the Hsp90 MD-CTD regions that stabilize the Hsp90 recognition interface. At the same time, Hsp90-kinase interactions protect a heterogeneous Cdk4 ensemble in a spatially confined inflated state. Distance fluctuation analysis also revealed differential destabilization (or polarization) of the kinase lobes, where a more dynamic N-lobe can be contrasted with more stable C-lobe. This effect was seen in the folded Cdk4-cyclin D complex, monomeric form of Cdk4, and in the Hsp90-Cdc37-Cdk4 complex (**Fig 5D**). These results are consistent with our recent studies in which we showed that divergences in the activation mechanisms of Cdk2 (nonclient) and Cdk4 (strong client) may be associated with differences in the relative stability and cooperativity of the kinase lobes in these Cdk proteins [**124**]. Interestingly, client binding can exploit differential stability of the kinase lobes by further increasing the disparity between highly dynamic N-lobe (tolerant to perturbations) and relatively stable C-lobe (more susceptible to perturbations). Conformational dynamics showed that the most flexible regions in the native Cdk4 structures correspond to the αC-β3 loop (46-NGGGGGGGL-54), β4 (residues 75–85), and β5 strands (residues 86–93) of the N-lobe. Strikingly, these regions are targeted and become partially unfolded upon recruitment to the Hsp90-Cdc37 chaperone. Our results suggested that the observed effect may reflect the intrinsic dynamic preferences of kinase clients, in which these N-lobe regions may be especially vulnerable to perturbations and form weak spots of kinase instability. These findings are consistent with similar effects seen in oncogenic kinases that are typically chaperone clients, where activation mutations are mainly assembled in a more flexible N-lobe that allows to readily modulating activation transitions [**125,126**]. Structural separation of stable and flexible regions in the chaperone-kinase complex highlight the notion that protein polarity can be required to afford evolution of new functions without sacrificing fold stability [**127–129**].

### Energetic analysis of protein-protein binding interfaces in the Hsp90-Cdc37-Cdk4 complex differentiates hotspot residues and quantifies functional role of the Cdc37-M/C domain

**Fig 6:**
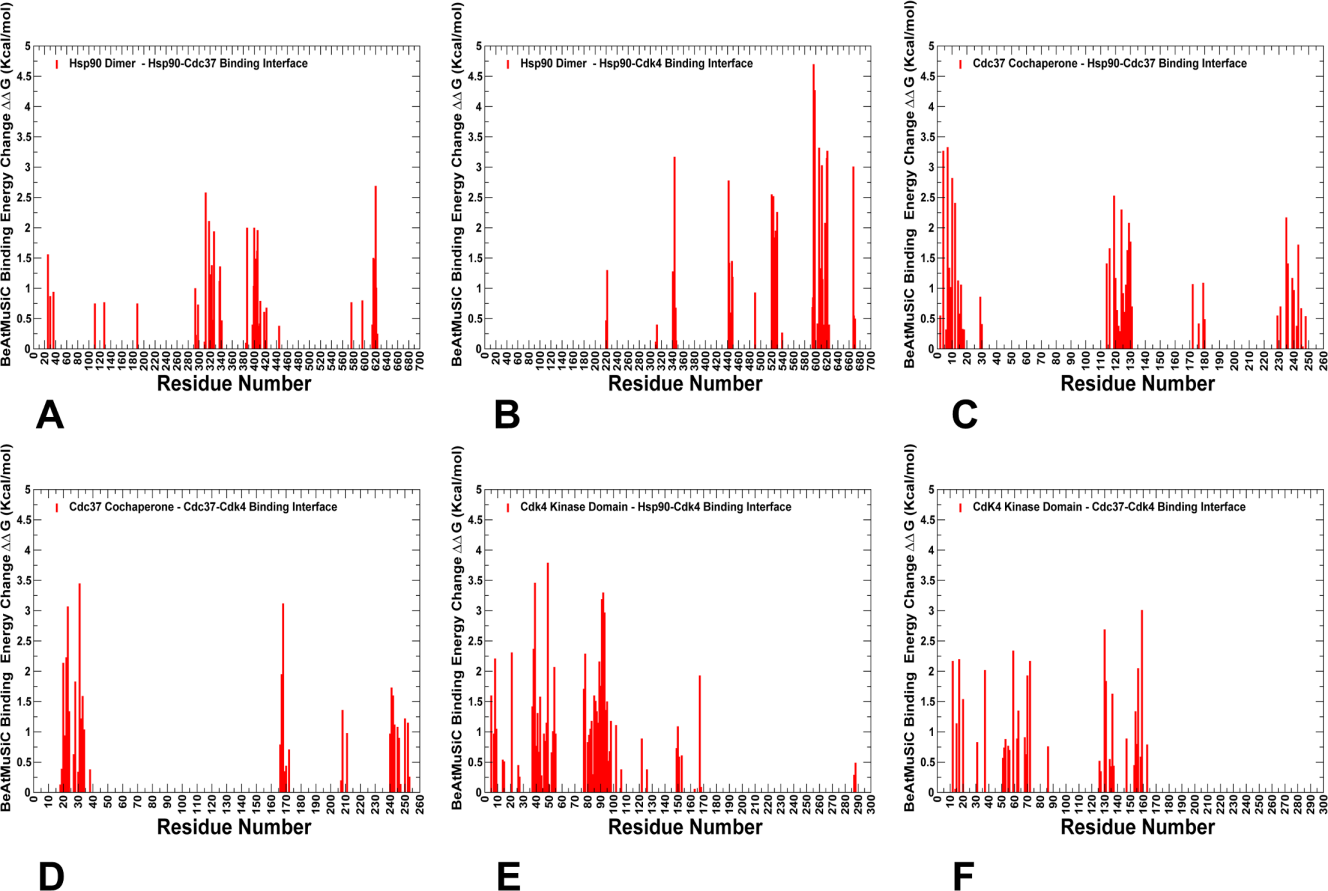
Alanine scanning and free energy analysis of the binding interfaces in the Hsp90-Cdc37-Cdk4 complex using BeAtMuSiC approach. Binding free energy changes obtained through alanine scanning of the interfacial residues in the Hsp90-Cdc37-Cdk4 complex using BeAtMuSiC approach. Mutation-induced binding free energy changes ΔΔG of the Hsp90 residues involved in the Hsp90-Cdc37 interface (A) and Hsp90-Cdk4 interface (B). Binding free energy changes ΔΔG of the Cdc37 residues forming interfacial contacts with Hsp90 (C) and Cdk4 (D). Binding free energy changes ΔΔG of the Cdk4 residues contributing to the Cdk4-Hsp90 interface (E) and Cdk4-Cdc37 interface (F). If the free energy change between a mutant and the wild type (WT) proteins ΔΔG = ΔG (MT)-ΔG (WT) > 0, the mutation is considered to be destabilizing. The distributions are shown in red-colored bars and highlight only binding interface residues.

**Fig 7:**
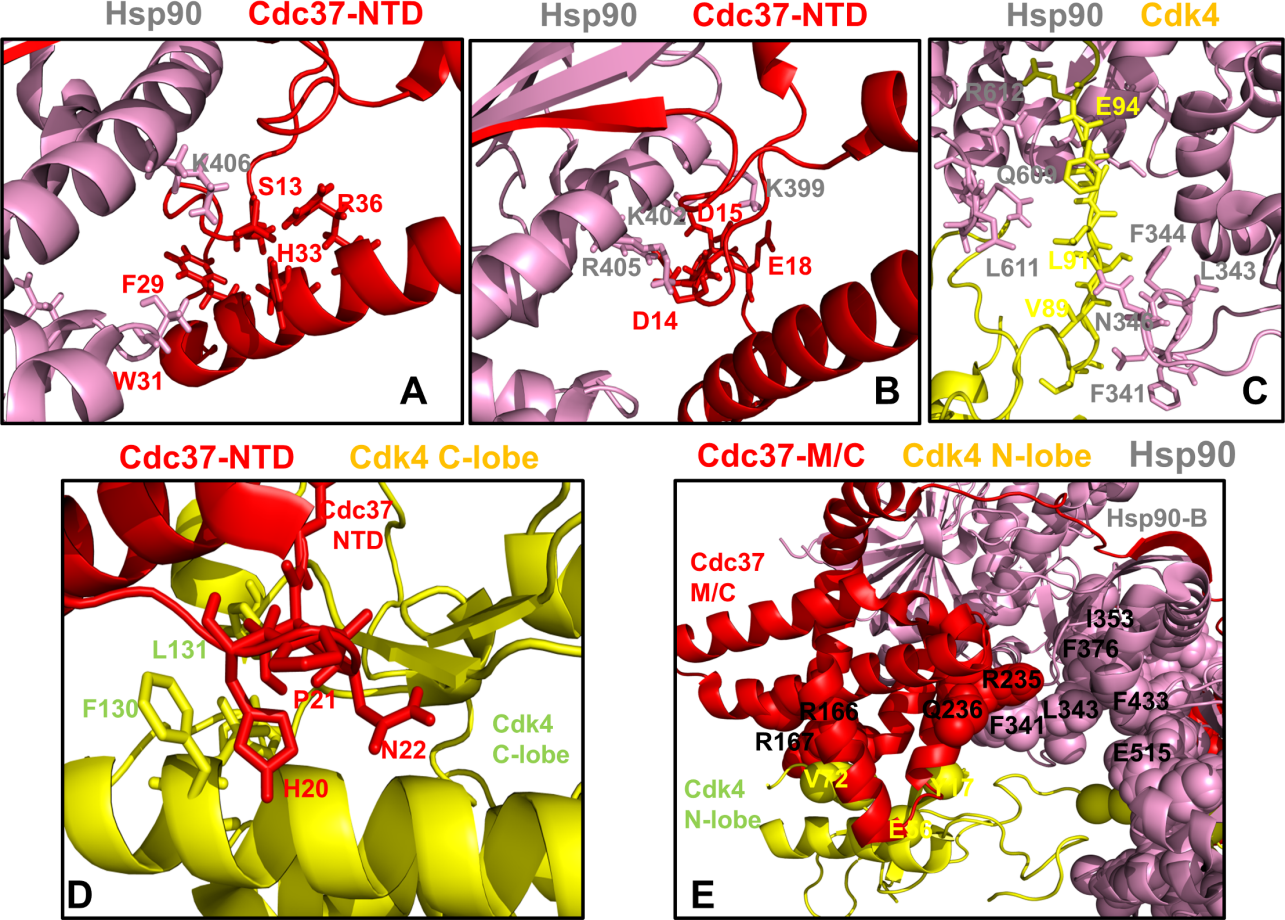
Structural mapping of the intermolecular interfaces and binding energy hotspots in the Hsp90-Cdc37-Cdk4 complex. Structural analysis of the binding energy hotspots and interfaces in the chaperone-kinase complex. (A) The Hsp90 binding interface with Cdc37-NTD is centered on phosphorylated S13 (Cdc37-NTD) forming electrostatic interactions with H33, R36 and Hsp90-K406. This interface involves several other Cdc37-NTD residues (F29 and W31). (B) Another Hsp90 interfacial network with Cdc37-NTD is formed through interactions of Cdc37-NTD residues (D14, D15, E16, D17, and E18) and Hsp90-MD residues (K399, K402, R405, and K406). This binding interface features six salt bridges that stabilize the Hsp90-Cdc37 interactions. The residues are shown in colored sticks according to the domain annotation. (C) The Hsp90 interface with the Cdk4 N-lobe highlights protection of the disordered region through interactions with the Hsp90-MD Src-loop (residues F341, L343, F344) and Hsp90-CTD residues L611 and M602. (D) The Cdc37-NTD binding interface with Cdk4 C-lobe features interactions between Cdc37-NTD hotspot segment 20-HPN-22 and C-lobe αF-helix residues F130, L131. In this interface the HPN motif mimics the shape and the interaction pattern of the αC-β4 loop (Cdk4 N-lobe) with the C-lobe. (E) The Cdc37-M/C interfacial hotspots (R245, Q246, R166, and R167) are involved in binding with Hsp90 and Cdk4 proteins. The residues are shown in sticks and colored according to the domain annotation (Cdc37-NTD in red, Hsp90-B in pink, Cdk4 in yellow).

We also performed alanine scanning of the binding interfaces in the Hsp90-Cdc37-Cdk4 complex by computing changes in protein-protein binding free energies induced by mutations of the interfacial residues (**Figs 6 and 7**). The central objective of this analysis was to determine energetic hotspots of protein binding and stability in the complex and quantify role of these functional residues in the allosteric mechanism of the chaperone-kinase cycle. In this analysis, we employed and compared three different computational methodologies that included coarse-grained, knowledge-based BeAtMuSiC predictor of binding free energy changes [**130–132**], empirical force field approach FoldX with the all-atom representation of protein structure [**133–137**], and the molecular mechanics Poisson–Boltzmann surface area (MM-PBSA) method [**138,139**], in which all-atom MD simulations were utilized to determine the ensemble-averaged changes in the binding affinity of the interacting proteins [**140–142**]. The knowledge-based BeAtMuSiC predictor utilizes a combination of 13 statistical potentials weighed by solvent accessibility and evaluates the effect of single missense mutations on the strength of interfacial interactions and the folding free energy of the complex [**130**]. We opted for this approach as a primary tool in our computations of mutation-induced binding free energy changes based on its known efficiency and robustness in reproducing experimental data. Indeed, BeAtMuSiC was amongst the top performing tools during community-wide evaluation of methods for predicting the effect of mutations on protein-protein interactions [**131**], where it produced a robust correlation with the experimental data from the SKEMPI database [**132**].

First, we present a detailed energetic analysis of the binding interfaces in the Hsp90-Cdc37-Cdk4 complex obtained from BeAtMuSiC predictions (**Fig 6**). The free energy profiles revealed a group of hotspot residues in the Hsp90-MD and Hsp90-CTD regions that contribute decisively to binding with Cdc37 (**Fig 6A**) and Cdk4 protein partners (**Fig 6B**). The Hsp90 binding interfaces with Cdc37 and Cdk4 are dominated by strong interactions of the Hsp90-MD hotspot residues (F341, L343, F344, K399, I400, L401, K402, K406) and Hsp90-CTD hotspots (M602, L611, R612) (**Fig 6A and 6B**). The hotspot residues often anchor local interaction clusters that are interconnected and stabilize binding interfaces in the complex (**Fig 7A and 7C**). One of these hydrophobic clusters (I404-V381-I408-L369-I370-I400-L401), which is centered around I400 and L401 hotspots, is linked with a group of interfacial lysine residues (K399, K402, K406, and K411) that form salt bridge interactions with Cdc37-NTD residues (**Fig 7A and 7B**). This cluster closely overlaps with an allosteric center in the Hsp90-MD (L369, I370, V381, L401, and I404). Another energetic hotspot is formed by cluster F376-L343-F433-N436 that anchored the Src-loop positions (R338, F341, L343, and F344) with the Cdk4 N-lobe (**Fig 7C**). Of particular significance was emergence of the Hsp90-CTD residues (M602, L611, R612) as prominent energetic hotspots of the Hsp90-Cdk4 binding interface. These observations corroborated with the dynamics analysis, showing that conformational flexibility of the Hsp90-CTD binding interface with Cdk4 was largely restricted due to favorable interactions made by these residues. A key energetic hotspot of the Cdc37 binding interface with Cdk4 corresponded to Cdc37-NTD motif 20-HPN-22 that mimics the shape and the interaction pattern of the αC-β4 loop (Cdk4 N-lobe) with the C-lobe (**Fig 7D**). According to our analysis, structural and energetic mimicry provided by the HPN motif may favor he displacement of the αC-β4 loop and Cdc37-induced stabilization of the lobe-separated, open kinase state. A strong energetic contribution to binding affinity was detected for several residues in the Cdc37-M/C domain (R166, R167 and Q208) (**Fig 6C and 6D**). Structural mapping of the Cdc37-M/C interfaces highlighted the decisive role of the Cdc37 hotspots (R245, Q246, R166, and R167) in binding with Hsp90 and Cdk4 respectively (**Fig 7E**). Interestingly, the predicted Cdc37-M/C hotspots R166, R167 and Q208 were previously reported as important interfacial sites in the NMR study of the complex between human Cdc37 (residues 148–276) and human Hsp90-NTD [**143,144**]. A biochemical analysis of the full-length human Hsp90-Cdc37 complex in living cells showed that mutations in the Cdc37 (M164A, R167A, L205A, and Q208A) could reduce the Hsp90-Cdc37 interactions by as much as 70–95%. [**145**]. in the context of our analysis, these residues may correspond to conserved hotspots that can be exploited by Cdc37 in binding with different partners and in different structural contexts. In the Hsp90-Cdk4 interface, several Cdk4 residues (K88, E94) from a partially disordered segment anchored strong electrostatic interactions with E345 (Hsp90-MD) and R612 (Hsp90-CTD) that can impose spatial constraints on the dynamic ensemble of open Cdk4 conformations (**Fig 6E and 6F**). We argue that spatial compaction of the heterogeneous client ensemble can speed up conformational search within the funnel of globule-like states and thereby facilitate process of client refolding and release from the chaperone system.

**Fig 8:**
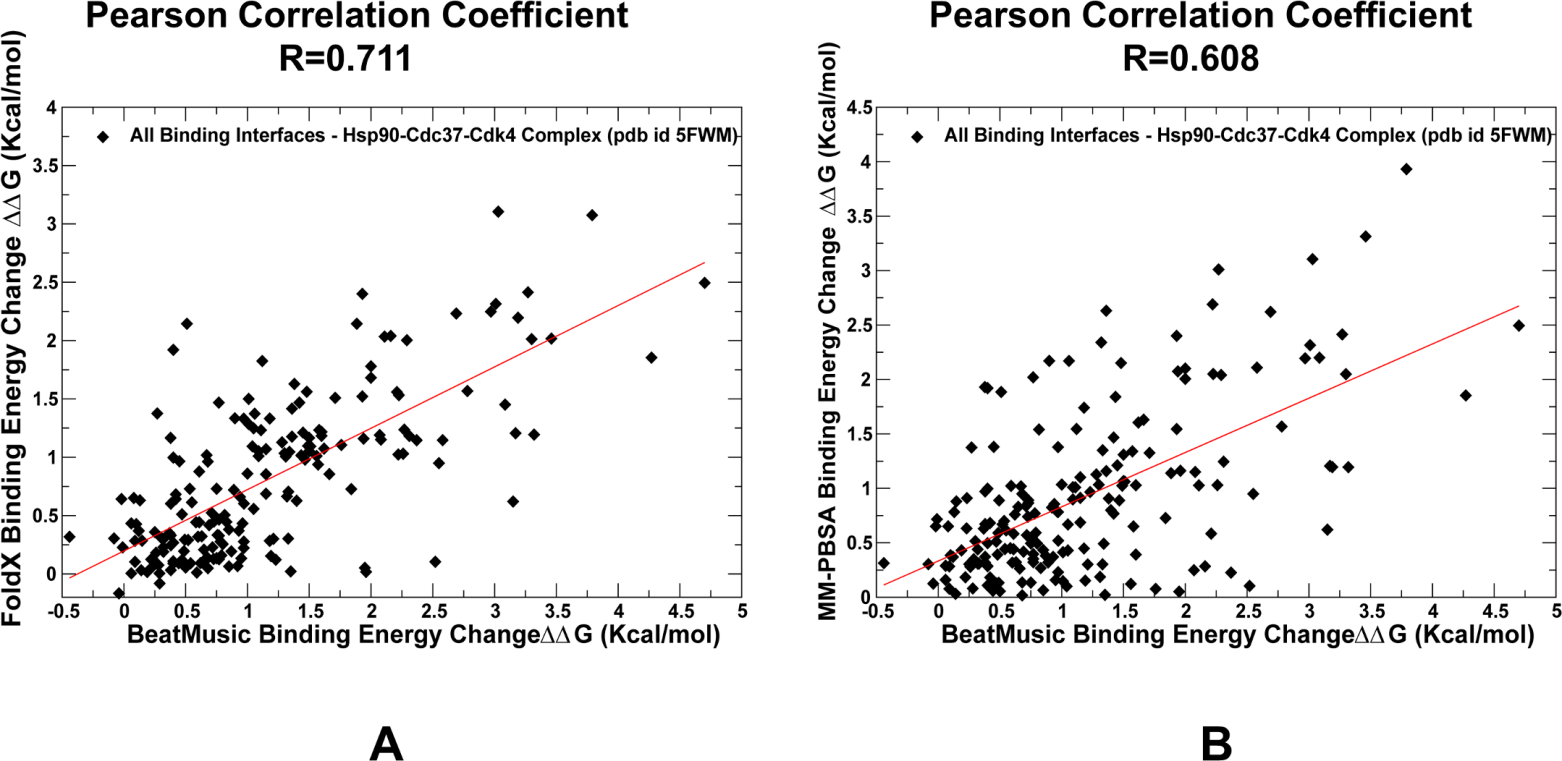
A comparison of predicted binding free energy changes induced by alanine mutations of the interfacial sites in the Hsp90-Cdc37-Cdk4 complex. A scatter plot of predicted binding free energy changes for all interfacial residues computed by BeAtMuSiC and FoldX methods (A). A similar scatter plot for binding free energy changes computed using BeAtMuSiC and MM-PBSA methods (B). The values of Pearson correlation coefficient are displayed on panels (A) and (B). The individual data points are shown as filled black diamonds.

Our results supported the notion that while Cdc37-NTD may control initial recognition and screening of client folds, a subsequent thermodynamic separation of strong and weak kinase clients may be determined through modulation of binding affinity by the Cdc37-M/C domain [**64,65**]. The energetic analysis of the Hsp90-Cdc37 interactions with the kinase client also supported a model in which binding may promote flexibility of the client substrate while simultaneously inducing stabilization of the flexible recognition regions in Hsp90. This mechanism may underlie allosteric cooperativity between Cdc37 domains and Hsp90 during recognition and recruitment of kinase clients. To further validate energetic predictions, we compared the BeAtMuSiC results (**Figs 6 and 7**) with the FoldX method (**Fig 8A**) and MM-PBSA approach (**Fig 8B**). The results displayed a strong positive correlation between binding free energy changes computed by different tools, also pointing to similar group of residues as energetic hotspots. Only a slightly lower correlation coefficient (R = 0.608) was found when BeAtMuSiC predictions were plotted against MM-PBSA results (**Fig 8B**). A somewhat greater dispersion of binding free energy changes in this comparison was likely due to the ensemble-average estimates in MM-PBSA calculations that were based on MD simulation trajectory of the complex. In general, a comparison of three different energetic methods produced consistent results, highlighting efficiency of knowledge-based BeAtMuSiC predictor in the analysis of binding free energies of protein-protein complexes. A detailed quantitative analysis of the binding interfaces in the Hsp90-Cdc37-Cdk4 complex obtained from MM-PBSA calculations (**S3 Fig**) demonstrated a close correspondence with BeAtMuSiC predictions. Noteworthy, these methods produced similar distribution peaks and predicted the same binding energy hotspots, suggesting that these salient signatures of the binding interfaces may be fairly robust and largely independent on the details of the energy force field. In particular, MM-PBSA calculations reaffirmed an important role of the Hsp90-CTD residues (M602, L611, R612) as key energetic hotspots of the Hsp90-Cdk4 binding interface (**S3 Fig**). Additionally, the MM-PBSA profiles highlighted a significant contribution of the Cdc37-M/C domain (R166, R167) in binding with Cdk4 client and strong energetic effect of the Hsp90 interactions with the disordered kinase region. In general, the energetic analysis quantified contribution of different binding interfaces and reinforced the notion that client recognition by the Hsp90-Cdc37 chaperone system may involve a significant dynamic and energetic exchange between binding partners near the extended hydrophobic interfaces.

### A model of chaperoning kinase clients by targeting weak spots of intrinsically dynamic kinase domains: Insights from network-based rigidity analysis and thermal unfolding simulations

Using FIRST approach [**146–150**] and the Python-based Constraint Network Analysis (CNA) interface [**151,152**] we also performed network-based rigidity decomposition of the Hsp90 and Cdk4 structures in different functional states. This analysis particularly quantified changes in the rigidity and flexibility of Cdk4 kinase domain in the unbound form, Cdk4-cyclin D complex and Hsp90-Cdc37-Cdk4 complex. The underlying hypothesis behind this approach is that a) differential stabilization of the N-lobe and C-lobe in the client kinase may be intrinsically present in the Cdk4-cyclin D complex and b) Hsp90-Cdc37 chaperone may exploit intrinsic flexibility of the N-lobe and presence or weak spots to amplify instability and induce a partially unfolded kinase state that is protected by the chaperone. In the FIRST approach, thermal unfolding of protein structures is emulated by gradually removing noncovalent constraints from the constrained network of bonds connecting residue nodes in the protein structure. This procedure can determine whether a bond is flexible or rigid and decompose the constraint network into rigid clusters and flexible regions. During unfolding, the weak constraints are removed first while stronger interactions are sustained longer, leading to progressive decomposition into rigid and flexible regions. We monitored the evolution of the ‘giant’ rigid cluster that disintegrates and breaks apart into a number of smaller rigid clusters during unfolding phase transition. Rigidity decomposition algorithm identifies unfolding nuclei as residues that break away from the giant rigid cluster near the transition point and become flexible. These sites are considered to be ‘weak spots’ that could initiate unfolding. The higher the frequency of a particular residue to be a weak spot, the greater the likelihood that the corresponding site corresponds to a highly flexible region. We incorporated MD-based ensembles of Cdk4 conformations in different states and used 500 representative samples for each of the trajectories to conduct thermal unfolding simulation on an ensemble of networks created from an ensemble of input structures [**151,152**]. Using this approach, the frequencies for all residues to become weak spots at the unfolding transition point were computed.

**Fig 9:**
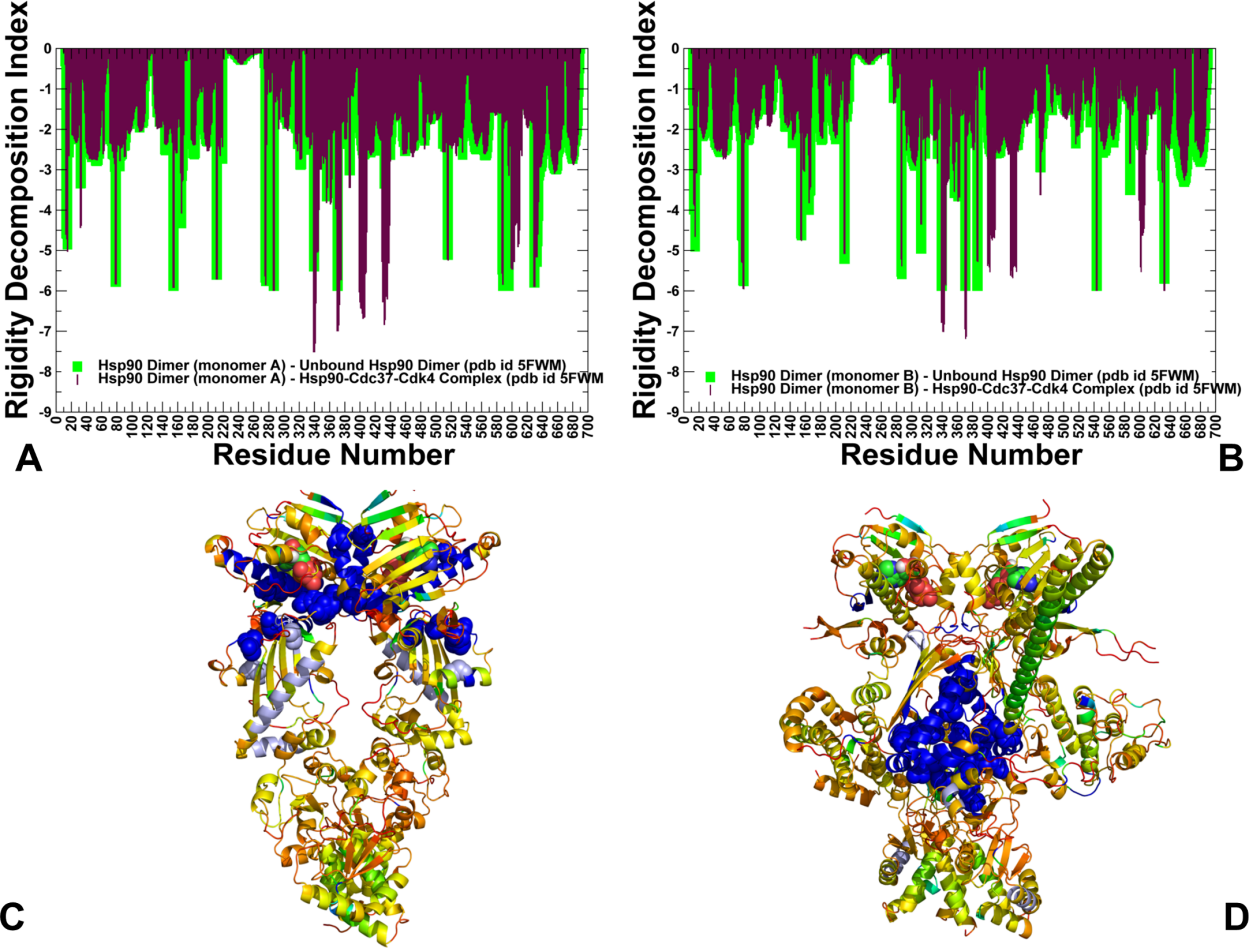
Rigidity decomposition analysis: Residue rigidity profiles in the unbound and client-bound Hsp90 forms. The rigidity index profiles of the Hsp90-monomer (A), Hsp90-B monomer (B). The rigidity indexes for the Hsp90 dimer in the apo form (green bars) an in the Hsp90-Cdc37-Cdk4 (maroon bars). Residue-based numbering of Hsp90 is the following: Hsp90-NTD (residues 1–215), Hsp90-MD (residues 216–552), and Hsp90-CTD (residues 553–690). Structural map of Hsp90 residue rigidity indexes in the unbound Hsp90 dimer (C) and Hsp90-Cdc37-Cdk4 complex (D). The color gradient from blue to red indicates the decreasing structural rigidity (or increasing conformational mobility) of the protein residues. Residues corresponding to the peaks in the rigidity index profiles are shown in spheres colored according to their rigidity level. ATP molecules are shown in atom-colored spheres.

The rigidity profile of the unbound Hsp90 dimer showed that the most stable residues resided in the ATP binding and near the NTD-MD interfaces (**Fig 9A and 9B**). The distribution peaks corresponded to the nucleotide binding site residues in the NTD (Y33, E42, L43) and catalytic loop in the Hsp90-MD (residues 382–402) with a particularly strong spike at a critical catalytic residue R392 (R380 in yeast Hsp90) that coordinates the γ-phosphate of ATP. Another peak corresponded to a hydrophobic cluster at the NTD-MD interface that is formed by residues W289, P287, F304, Y356 and F208. This interaction cluster is conserved in the Hsp90 proteins and structurally proximal to another group of rigid sites in the Hsp90 dimer (L388, I390 and R392). Together, these clusters of rigid residues are responsible for cross-subunit NTD interactions and stabilization of the catalytic site environment [**123**]. Notably, the most rigid regions in the unbound Hsp90 dimer are assembled near the nucleotide binding site and the NTD-MD interface (**Fig 9A and 9B**). Structural mapping of highly stable sites highlighted decomposition of rigidity and flexibility in the Hsp90 dimer (**Fig 9C**) and Hsp90-Cdc37-Cdk4 complex (**Fig 9D**). In the Hsp90 dimer, the distribution of rigid residues near the inter-domain interfaces reflected stabilization of the nucleotide binding site, coordinated dimerization of the Hsp90-NTDs and association of the Hsp90-NTD with the Hsp90-MD. Central to this analysis is the global propagation of rigidity in the chaperone complex, where the rigidity of the NTD regions was reduced, while and structural environment in the MD-CTD inter-domain regions was considerably rigidified (**Fig 9B**). During chaperone cycle, upon ATP hydrolysis, Hsp90 dimer is expected to open NTDs with Cdc37-Cdk4 still bound–a necessary step that enables kinase to refold before leaving the complex [**65, 69**].

**Fig 10:**
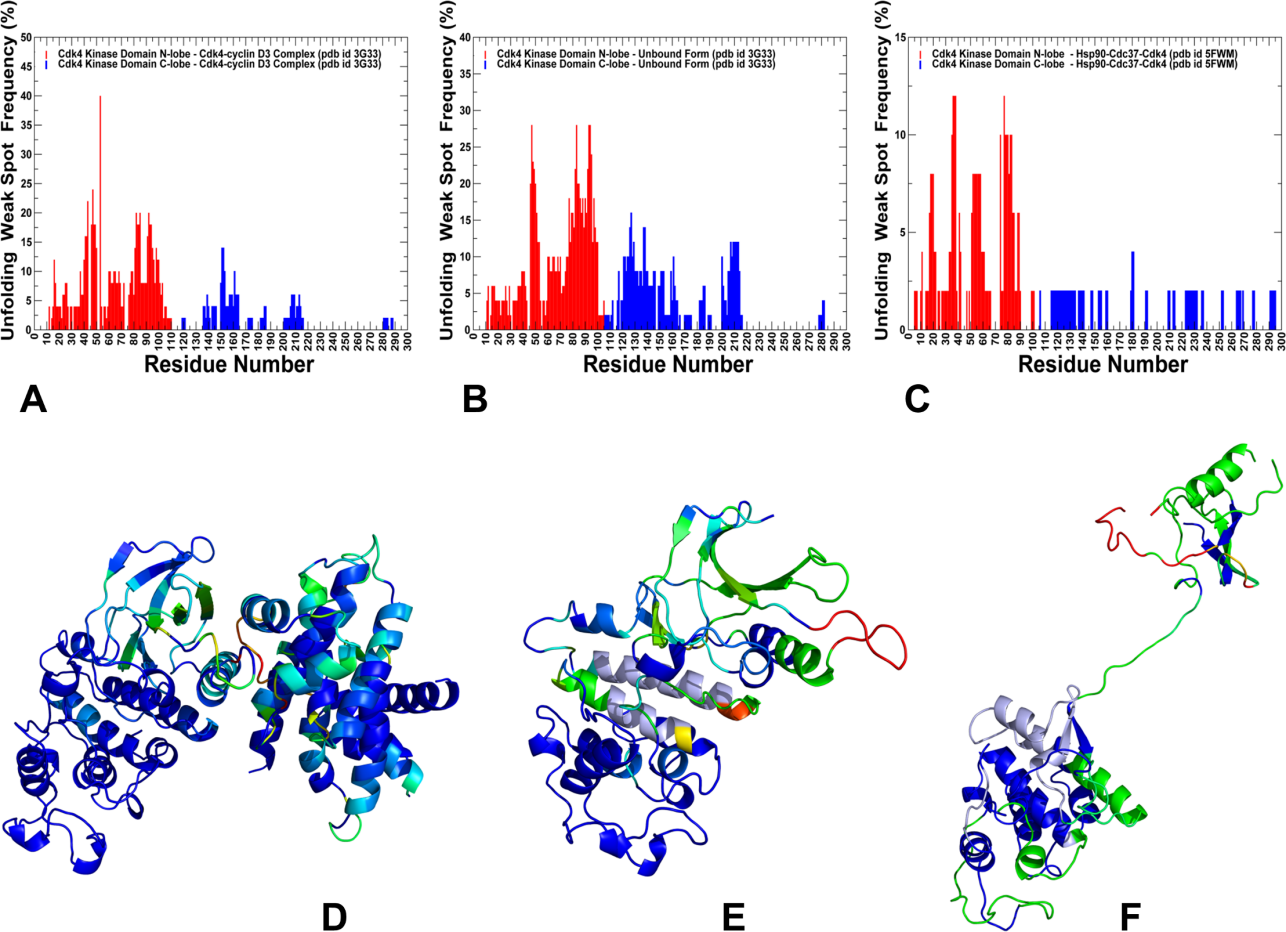
Rigidity decomposition analysis: Distribution of unfolding weak spots in the Cdk4 structures. The frequency of unfolding nuclei (weak spots) in the Cdk4 catalytic domain obtained from simulations of the Cdk4-cyclin D3 complex (A), monomeric unbound form of Cdk4 (B) and Hsp90-Cdc37-Cdk4 complex (C). The frequencies of the kinase domain residues are shown in colored bars, with the N-lobe residues in red bars and C-lobe in blue bars. Unfolding nuclei or weak spots are defined as residues that break away from the giant rigid cluster immediately after transition during the network-based emulation of thermal unfolding. The higher the frequency of a weak spot, the more probable unfolding begins from these residues. Structural maps of rigidity/flexibility regions in the Cdk4 catalytic domain at the unfolding transition point obtained from emulation of thermal unfolding for the Cdk4-cyclin D3 complex (D), the monomeric unbound Cdk4 form (E) and the Hsp90-Cdc37-Cdk4 complex (F). Cdk4 conformations are colored using a color range from red (highest ranking weak spot) to blue (lowest ranking weak spot).

We monitored changes in the rigidity and flexibility of Cdk4 client (**Fig 10**) in the Cdk4-cyclin D3 complex, Cdk4 unbound form and in the Hsp90-Cdc37-Cdk4 complex. Strikingly, even in the complex with cyclin, we observed polarization of kinase lobes, with a mostly rigid C-lobe and a more dynamic N-lobe. The weak spots in the Cdk4-cyclin D3 complex and Cdk4 unbound form corresponded to the intrinsically flexible β3-αC loop and β4-β5 strand regions (**Fig 10A and 10B**). These regions are targeted by the Hsp90-Cdc37 and become partially unfolded in the Hsp90-Cdc37-Cdk4 (**Fig 10C**). The β3-αC loop plays an important role in modulating dynamics of the adjacent regulatory αC-helix, thus exerting tight control over kinase activity [**153**]. According to our findings, these N-lobe regions may serve as initiation sites for kinase unfolding and present weak spots of kinase instability that are targeted by the chaperone. Structural mapping of unfolding nuclei residues showed the evolution and gradual expansion of the weak spot clusters (**Fig 10D and 10F**). Of special significance was the emergence of localized weak spots in the Cdk4-cyclin D3 complex, where the β3-αC loop and β4-β5 were the most flexible regions (**Fig 10D**). In the cyclin-free, monomeric form of Cdk4, we observed a gradual expansion of the weak spot regions (**Fig 10E**), where large fractions of the N-lobe formed flexible clusters, including the G-loop, β3-αC loop, β4-β5, and β6-β7 strands. Interestingly, in the monomeric Cdk4 form, the entire layered β-sheet region of the N-lobe becomes flexible near the unfolding transition point. It is worth noting that the instability of the β-sheet regions is believed to be a common dynamic characteristic shared by many kinase clients of the Hsp90-Cdc37 chaperone [**60**]. In all functional forms of Cdk4, the distribution of rigid and flexible regions is not uniform as the C-lobe harbors most of the rigid residues, while the N-lobe residues belonged to flexible regions (**Fig 10D and 10F**). The important finding of this analysis is evidence for differential stabilization of the kinase lobes in Cdk4 client and the emergence of the primary unfolding weak spot near the β3-αC loop and β4-β5 strand of the N-lobe. These characteristic features of the Cdk4 client may be intrinsically present in different functional forms of the kinase client. Consistent with the cryo-electron microscopy structure of the Hsp90-Cdc37-Cdk4 kinase complex [**69**], network emulation of thermal unfolding showed that these segments would correspond to the weak spots of Cdk4 client. We argue that Cdc37 and Hsp90 can recognize and exploit this signature of the kinase client by targeting the predicted weak spots of kinase instability to unfold and trap the N-lobe of Cdk4 during kinase recruitment.

Our results suggest a mechanism for client kinase recognition by the Hsp90-Cdc37 machine. We propose that differential stabilization of kinase lobes is sensed by Cdc37 cochaperone that initially binds and splits kinase at the lobes, creating platform for Hsp90 intervention. At the next stage, weak spots of the N-lobe, that are most vulnerable to structural perturbations in the dynamic kinase client, can be recognized by the Hsp90 binding site regions. Through a cross-talk between Hsp90 and Cdc37 binding interfaces, the chaperone system effectively traps partially unfolded kinase conformations and allow for conformational remodeling of the client that is initiated from the weak spot sites. The bound clients are held by chaperones in a spatially confined dynamic ensemble, which may increase the chances of refolding by facilitating intramolecular interactions. According to our findings, robust recognition of weak kinase spots and reciprocal dynamic exchange at the Hp90-kinase interface may represent a plausible mechanistic scenario underlying chaperone-kinase interactions. In the proposed model of chaperone-kinase functional cycle, allosteric interactions between Hsp90 and Cdc37 may facilitate conformational and energetic polarization of the kinase lobes, inducing partial unfolding that is thermodynamically controlled through an entropy transfer between Hsp90 and client kinase. This mechanism may underlie allosteric cooperativity Cdc37 and Hsp90 during initial screening of client folds and subsequent thermodynamic sorting of strong and weak clients, which may be controlled by the Hsp90. The results of this study may help to explain how allosterically regulated machines such as Hsp90-Cdc37 can fulfill their chaperoning function by selectively remodeling the folding energy landscapes of their diverse protein clients.

## Materials and methods

### MD simulations

All-atom MD simulations have been performed for the structure of the human Hsp90-Cdc37-Cdk4 complex (pdb id 5FWM, 5FWK, 5FWL) [**69**], Cdk4/cyclin D1 complex (PDB 2W9FZ) [**98**], Cdk4/cyclin D3 complex (PDB 3G33) [**99**]. We carried out five independent 500 ns for the human Hsp90-Cdc37-Cdk4 complex and ten independent 500 ns for the Cdk4-cyclin D complexes. The conformational ensembles and computed properties of the complexes were generated using aggregation of all independent trajectories. All structures were obtained from the Protein Data Bank [**154**]. Structure preparation process included several main steps previously reported in detail in our studies of molecular chaperones [**155,156**]. In brief, in this protocol, hydrogen atoms, missing residues and protonation states were assigned using the WHATIF program [**157**]. The missing segments of in the Hsp90-Cdc37-Cdk4 complex and the kinase crystal structures were modeled using the ArchPRED program [**158**]. MD simulations were performed using CHARMM22 force field in the NAMD 2.6 package [**159**]. The simulation steps and specifics of the MD protocol were fully consistent with the workflow previously detailed in our studies of molecular chaperones [**155,156**]. In brief, the following protocol preceded the 500 ns production stage of simulations. All atoms of the complex were first restrained at their crystal structure positions with a force constant of 10 Kcal*mol^-1^*Å^-2^. The system was subjected to the following simulation annealing to ensure the proper equilibration. The temperature was increased from 0K to 500K at a rate of 1K per 1ps and was kept at 500K for 500ps. The temperature was then decreased from 500 K to 300K at a rate of 1K per 1ps and was kept at 300K for additional 500ps. An NPT production simulation was then run on each of the equilibrated structures for 500 ns keeping the temperature at 300 K and constant pressure of 1 atm.

### Distance fluctuations analysis

In this protein mechanics-based approach [**121–123**], we evaluated residue stability and flexibility through distance fluctuation analysis of the conformational ensembles for studied systems. The fluctuations of the mean distance between a given residue and all other residues in the ensemble were converted into residue stability indexes that measured the energy cost of the residue deformation during simulations. In this model, the high stability indexes would be associated with residues that display small fluctuations in their distances to all other residues, while small values of this stability parameter would point to more flexible sites that experience large deviations of their inter-residue distances. We computed the fluctuations of the mean distance between each atom within a given residue and the atoms that belong to the remaining residues of the protein. The force constant for each residue is computed as the average of the force constants for all its atoms. Alternatively, the mean fluctuations of a given residue can be also characterized using only *C*_*α*_ atom positions. In our model, the force constant for each residue is calculated by averaging the distances between the residues over the MD trajectory using the following expression:

$$k_{i}=\frac{3k_{B}T}{\left\langle {\left(d_{i}-\left\langle d_{i}\right\rangle \right)}^{2}\right\rangle }$$ (1)

$$d_{i}={\left\langle d_{ij}\right\rangle }_{j^{*}}$$ (2)

*d*_*ij*_ is the instantaneous distance between residue *i* and residue *j*, *k*_*B*_ is the Boltzmann constant, *T* = 300K. ⟨ ⟩ denotes an average taken over the MD simulation trajectory and $d_{i}={\left\langle d_{ij}\right\rangle }_{j^{*}}$ is the average distance from residue *i* to all other atoms *j* in the protein. The sum over j_*_ implies the exclusion of the atoms that belong to the residue *i*. The interactions between the *C*_*α*_ atom of residue *i* and the *C*_*α*_ atom of the neighboring residues *i*−1 and *i*+1 are excluded in the calculation since the corresponding distances are nearly constant. The inverse of these fluctuations yields an effective force constant *k*_*i*_ that describes the ease of moving an atom with respect to the protein structure. The average distance fluctuations profiles defined residue stability indexes and characterized the distribution of stable and flexible regions in the protein structures. Allosteric communication propensities of protein residues are associated with their mechanistic properties and mean square deformations of the inter-residue distances that could be related to the inter-residue commute time [**87–90**]. In this model, an efficient allosteric communication proceeds through coupling of spatially separated rigid hinge sites that transmit allosteric structural changes.

### Protein-protein binding free energy calculations

We employed three independent approaches to compute protein-protein binding energy changes induced by alanine mutations of the interfacial residues in the Hsp90-Cdc37-Cdk4 complex. These approached included coarse-grained BeAtMuSiC predictor of binding free energy changes [**130–132**], empirical force field approach FoldX with the all-atom representation of protein structure [**133–137**], and MM-PBSA method [**138–142**] that utilizes all-atom MD simulations. In these models, the binding free energy of protein-protein complex is expressed as difference in the folding free energy of the complex and folding free energies of the two protein binding partners:

$$\Delta G_{bind}=G^{com}-G^{A}-G^{B}$$ (3)

The change of the binding energy due to a mutation can be calculated then as the following:

$$\Delta \Delta G_{bind}=\Delta G_{bind}^{mut}-\Delta G_{bind}^{wt}{{}_{}}^{}$$ (4)

The BeatMusic approach employs a combination of statistical potentials that describe pairwise inter-residue distances, backbone torsion angles and solvent accessibilities derived from known protein structures [**160**]. In this model, the change in the binding free energy of protein-protein complex upon mutation is expressed as follows:

$$\Delta \Delta G_{bind}=\sum_{i=1}^{13}\alpha (\mathrm{SA})\Delta \Delta W_{i}+\alpha_{14}(\mathrm{SA})\Delta V_{+}+\alpha_{15}(\mathrm{SA})\Delta V_{-}+\alpha_{16}(\mathrm{SA})$$ (5)

where ΔΔ*W*_*i*_ describe the free energy change induced by 13 different statistical potentials, the terms Δ*V*_+_ and Δ*V*_−_ account for the packing defects, and the weights *α*_*i*_(SA) are empirical functions of the solvent accessibility of the mutated residue identified from experimental folding free energy changes induced by mutations [**161**].

To obtain ensemble-based estimates of binding free energy changes and permit multiple runs of BeAtMuSiC predictor for independent conformations from MD trajectory, we employed Apache Jmeter open source software Java application (http://jmeter.apache.org) designed to simulate multiple requests to a target server from a user (or group of users). This software is used in bioinformatics applications to configure test plans and perform load testing for batches of multiple server requests, store and save all responses, and calculate statistics [**162**]. A number of threads were initiated by Apache Jmeter application to process multiple BeAtMuSiC predictor requests to the server, store and analyze results and assess server performance.

Alanine scanning of protein binding interface residues in the Hsp90-Cdc37-Cdk4 structure was also performed using FoldX approach [**133–137**]. In this model, the structure of the WT Hsp90-Cdc37-Cdk4 complex was optimized by the RepairPDB function within FoldX [**136**]. The conformations of the alanine mutant complexes were generated from the optimized WT structures by the BuildModel function within FoldX. To calculate binding free energies by FoldX, we used Eq (3) and calculated the folding free energy of the Hsp90-Cdc37-Cdk4 complex and each protein separately for the mutant and WT structures. The change of the binding energy due to a mutation is then calculated according to Eq (4).

Binding energies were also calculated based on the MM-PBSA method that combines the molecular mechanics force field with the Poisson–Boltzmann continuum representation of the solvent [**138–142**]. The total free energy Δ*G*_*bind*_ in MM-PBSA is expressed as the sum of the gas phase contribution Δ*G*_*MM*_, the solvation free energy Δ*G*_*solv*_, and an entropic contribution −*T*Δ*S*:

$$\Delta G_{bind}=<\Delta G_{MM}>+<\Delta G_{solv}>-T\Delta S$$ (6)

The brackets <> denote an average of these contributions calculated over the MD trajectories. The gas-phase contribution <Δ*G*_*MM*_> to the binding free energy is the difference in the molecular mechanics energy of the complex and the isolated proteins. This contribution is the sum of the differences in the internal energies Δ*E*_intra_, the van der Waals interaction energy Δ*E*_*vdw*_, and the electrostatic interaction energy Δ*E*_*elec*_:

$$<\Delta G_{MM}>=\Delta E_{\mathrm{intra}}+\Delta E_{vdw}+\Delta E_{elec}$$ (7)

$$E_{\mathrm{intra}}=E_{bond}+E_{vdw}+E_{elec}$$ (8)

where *E*_*bond*_ is the energy of the bonded terms (bonds, angles, dihedral angles, and improper angles) of a given molecule; *E*_*vdw*_ is the van der Waals energy of the molecule; and *E*_*elec*_ is the electrostatic energy of the molecule. These contributions are calculated according to the CHARMM22 force field and describe the molecular mechanical energy in the gas-phase.

The solvation free energy Δ*G*_*solv*_ is the difference between the solvation energy of the complex and solvation free energies of the isolated protein and ligand:

$$\Delta G_{solv}=G_{solv}^{complex}-G_{solv}^{A}-G_{solv}^{B}$$ (9)

$$\Delta G_{solv}=\Delta G_{solv}^{np}+\Delta G_{solv}^{pol}$$ (10)

All energy terms are calculated using MD trajectories of the Hsp90-Cdc37-Cdk4 complex, which is followed by separation of the complexes into individual protein structures and subsequent minimization of the isolated molecules. All energy terms were calculated for 1,000 frames regularly separated by 500 ps along the 500ns trajectory performed for the complex. Due to large size of the studied system and presence of partially disordered Cdk4 protein in the complex, the entropy calculations are very computationally demanding and can be prone to significant statistical errors [**142**]. The entropy terms for the binding free energy changes may also cancel each other later assuming that protein structures do not experience significant rearrangements upon mutations. Based on these considerations, the entropy calculations were not performed in our binding free energy calculations.

### Network-based modeling of protein rigidity and thermal unfolding

We utilized FIRST (Floppy Inclusion and Rigid Substructure Topography) approach [**146–150**] and the Python-based Constraint Network Analysis (CNA) interfaces [**151,152**] to build a network of the covalent and noncovalent bond constraints in the protein. In the FIRST approach, hydrogen bonds, salt bridges, and hydrophobic contacts define noncovalent bond constraints that are calculated with an empirical energy function. A hydrogen bond energy *E*_*hb*_ is calculated using a geometry-based empirical function [**163**] and only hydrogen bonds with an energy below cutoff *E*_*cuthb*_ = -1.0 kcal/mol are included in the network. Hydrophobic contacts are considered between all carbon and sulfur atoms separated by a distance less than the sum of their van der Waals radii (1.7 Å for C and 1.8 Å for S) plus a temperature-independent *D*_*cuthp*_ = 0.25 Å. In the FIRST approach, rigidity changes are monitored by a gradual removal of hydrogen bonds in the order of increasing strength, keeping all covalent and hydrophobic interactions and repeating the rigidity analysis at each step, thus decomposing protein structure into rigid and flexible regions. This algorithm determines whether a bond is flexible or rigid and decomposes the constraint network into rigid clusters and flexible regions. A rigid cluster is a set of residue nodes that move together as a rigid body, whereas residues that are not a component of a rigid cluster are assigned to a flexible region. During this process, the weak constraints are removed first while stronger interactions are sustained longer, leading to progressive decomposition into rigid and flexible regions. Using this model, the rigidity index (*r*_*i*_) is calculated for each residue, which is defined by the energy value *E*_*cuthb*_ when the noncovalent constraints that involve a given residue are removed from any rigid cluster of the set of rigid clusters. In *C*_*α*_ atom-based representation of a residue adopted here, the average of the two *r*_*i*_ values of the two backbone bonds is taken. In this model, the lower the index value *r*_*i*_ the longer a given residue stays as a part of a rigid cluster during the thermal unfolding simulation, which is defining characteristic of residue rigidity. Thermal unfolding in the FIRST approach is implemented by emulating temperature-dependent unfolding trajectories. During unfolding, non-covalent constraints corresponding to weaker interactions that dissolve at low temperatures are removed from the network first, and each new network is then again decomposed into rigid and flexible clusters. By proceeding from a rigid network at low temperature to a flexible network at high temperature, unfolding phase transitions can be observed, at which point a giant rigid cluster in the network breaks apart into smaller rigid clusters. The identification of weak spots is performed using the CNA software package [**151,152**]. In this procedure, rigid cluster decompositions immediately before and after folded-unfolded transition are compared, and residues whose *C*_*α*_ atoms are part of the giant cluster before the transition, and leave the giant cluster after transition are identified as locally weak spots in the constraint network. A residue is considered flexible if its *C*_*α*_ atom is either in a flexible region or part of a small rigid cluster of less than four atoms. We employed MD-based ensembles of Cdk4 conformations obtained from multiple simulations of the Cdk4-cyclin D3 complex, unbound Cdk4 form, and Hsp90-Cdc37-Cdk4 complex. 500 representative samples for each of the trajectories were used to perform thermal unfolding simulation on an ensemble of networks created from an ensemble of input structures [**151,152**]. The frequencies of Cdk4 residues to become weak spots are computed and averaged over conformational ensembles for each of these functional forms. The frequency of all residues being predicted as a weak spot throughout the ensemble is counted and, finally, all weak spots are assigned a rank according to the decreasing order of their frequency.

## Supporting information

S1 Fig Structural mapping of hinge clusters in the Hsp90-Cdc37-Cdk4 complex. The hinge clusters are mapped onto the conformational mobility profiles along the slow collective modes. Two alternative 180º views of the Hsp90-Cdc37-Cdk4 structure are presented (A,B). Conformational dynamics profiles were computed by averaging protein motions in the space of five lowest frequency modes. The hinge residues are shown in spheres and colored according to their mobility in the slow modes. The color gradient from blue to red indicates the decreasing structural rigidity (or increasing conformational mobility) of the protein residues. (C) The hinge cluster near the NTD-MD inter-domain region is formed by conserved residues P287, W289, P287, F304, Y356 and F208 residues. A close proximity of this hinge center to the catalytic residue R392 and ATP is highlighted. R392 and ATP are shown in atom-based colored spheres. (D) The hinge clusters formed in the central core of the Hsp90-MD: F376-L343-F433-N436 and L369-I370-V381-L401-I404-V381. (E) The hinge centers near the MD-CTD interface are formed by clusters W598-M606-R612 and L455-I441-C521-L525-L533.
(TIF)

S2 Fig The residue depth analysis of the Hsp90-Cdc37-Cdk4 complex. The residue depth profiles of the Hsp90-A monomer (A), Hsp90-B monomer (B), Cdc37 (C) and Cdk4 proteins (D). The residue depth values are obtained from MD-based averaging over the conformational ensemble. The distributions are shown in domain-colored bars. The Hsp90-NTD (residues 1–215) is colored in red bars, Hsp90-MD (residues 216–552) is in blue bars, and Hsp90-CTD (residues 553–690) is in green bars. The Cdc37-NTD (residues 1–147) are colored in red bars, The Cdc37-M/C domain residues (residues 148–260) are in blue bars. The Cdk4 N-lobe (residues 1–99) is in red bars and Cdk4 C-lobe (residues 100–295) is in blue bars.
(TIF)

S3 Fig Alanine scanning and free energy analysis of the binding interfaces in the Hsp90-Cdc37-Cdk4 complex using MM-PBSA approach. Binding free energy changes obtained through alanine scanning of the interfacial residues in the Hsp90-Cdc37-Cdk4 complex using MM-PBSA approach. Mutation-induced binding free energy changes ΔΔG of the Hsp90 residues involved in the Hsp90-Cdc37 interface (A) and Hsp90-Cdk4 interface (B). Binding free energy changes ΔΔG of the Cdc37 residues forming interfacial contacts with Hsp90 (C) and Cdk4 (D). Binding free energy changes ΔΔG of the Cdk4 residues contributing to the Cdk4-Hsp90 interface (E) and Cdk4-Cdc37 interface (F). If the free energy change between a mutant and the wild type (WT) proteins ΔΔG = ΔG (MT)-ΔG (WT) > 0, the mutation is considered to be destabilizing. The distributions are shown in red-colored bars and highlight only binding interface residues.
(TIF)
