# Supplementary figures and images for: Atomistic simulations and network-based modeling of the Hsp90-Cdc37 chaperone binding with Cdk4 client protein: A mechanism of chaperoning kinase clients by exploiting weak spots of intrinsically dynamic kinase domains
Source: PLoS One. 2017 Dec 21;12(12):e0190267. doi: 10.1371/journal.pone.0190267 (PMC5739471; doi:10.1371/journal.pone.0190267)

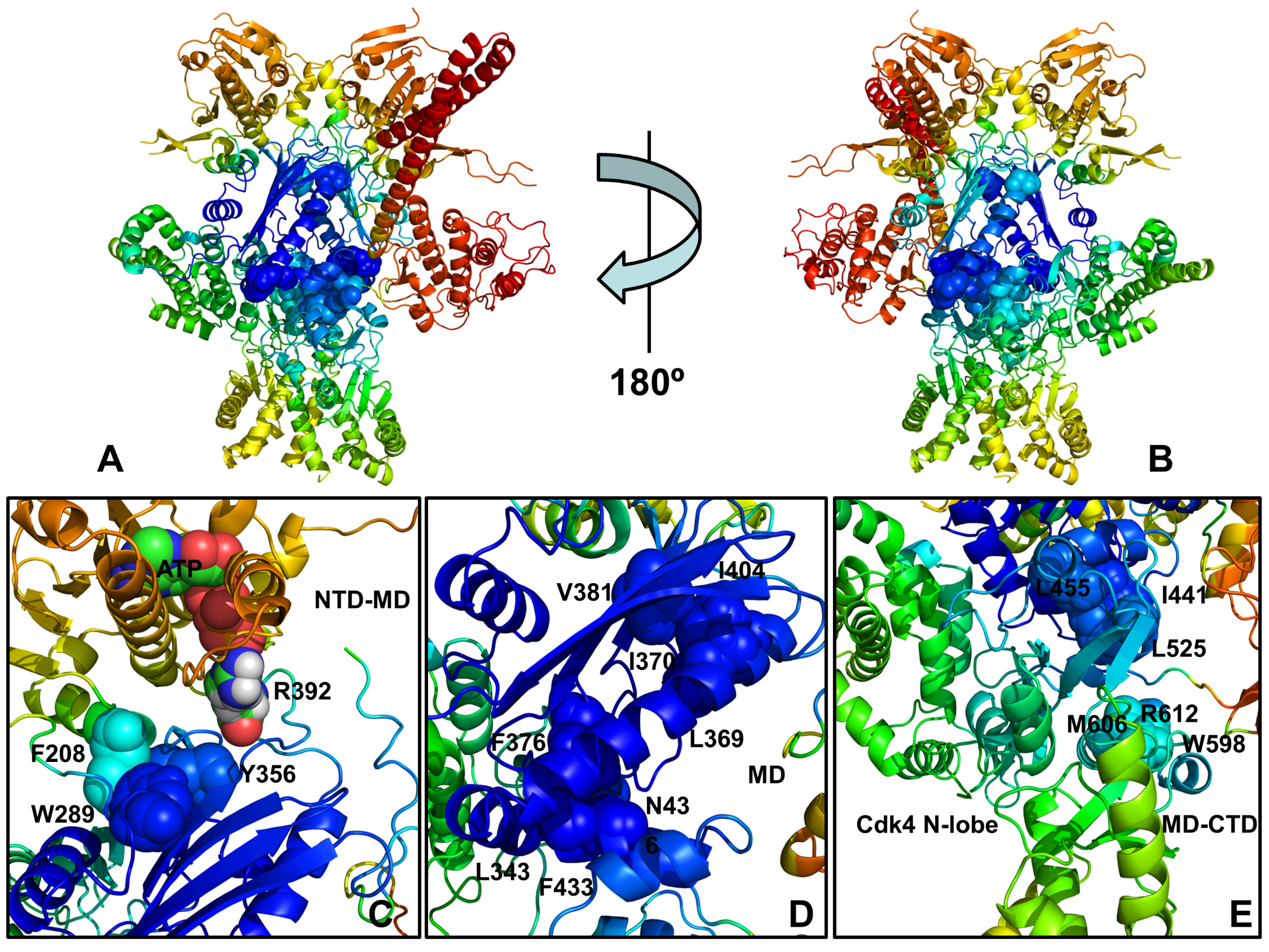

Supplement: S1 Fig — The hinge clusters are mapped onto the conformational mobility profiles along the slow collective modes. Two alternative 180º views of the Hsp90-Cdc37-Cdk4 structure are presented (A,B). Conformational dynamics profiles were computed by averaging protein motions in the space of five lowest frequency modes. The hinge residues are shown in spheres and colored according to their mobility in the slow modes. The color gradient from blue to red indicates the decreasing structural rigidity (or increasing conformational mobility) of the protein residues. (C) The hinge cluster near the NTD-MD inter-domain region is formed by conserved residues P287, W289, P287, F304, Y356 and F208 residues. A close proximity of this hinge center to the catalytic residue R392 and ATP is highlighted. R392 and ATP are shown in atom-based colored spheres. (D) The hinge clusters formed in the central core of the Hsp90-MD: F376-L343-F433-N436 and L369-I370-V381-L401-I404-V381. (E) The hinge centers near the MD-CTD interface are formed by clusters W598-M606-R612 and L455-I441-C521-L525-L533. (TIF) [file pone.0190267.s001.tif]

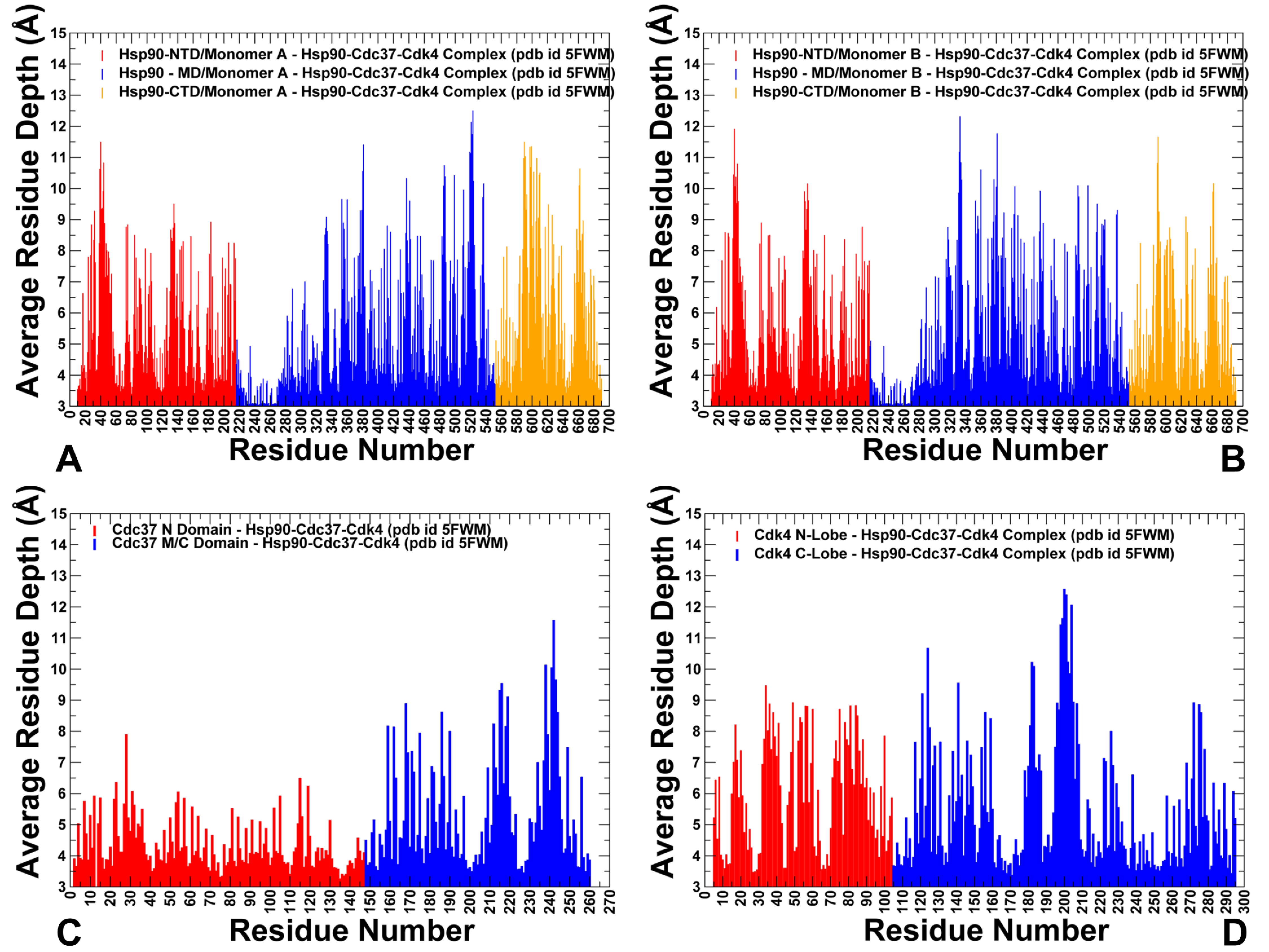

Supplement: S2 Fig — The residue depth profiles of the Hsp90-A monomer (A), Hsp90-B monomer (B), Cdc37 (C) and Cdk4 proteins (D). The residue depth values are obtained from MD-based averaging over the conformational ensemble. The distributions are shown in domain-colored bars. The Hsp90-NTD (residues 1–215) is colored in red bars, Hsp90-MD (residues 216–552) is in blue bars, and Hsp90-CTD (residues 553–690) is in green bars. The Cdc37-NTD (residues 1–147) are colored in red bars, The Cdc37-M/C domain residues (residues 148–260) are in blue bars. The Cdk4 N-lobe (residues 1–99) is in red bars and Cdk4 C-lobe (residues 100–295) is in blue bars. (TIF) [file pone.0190267.s002.tif]

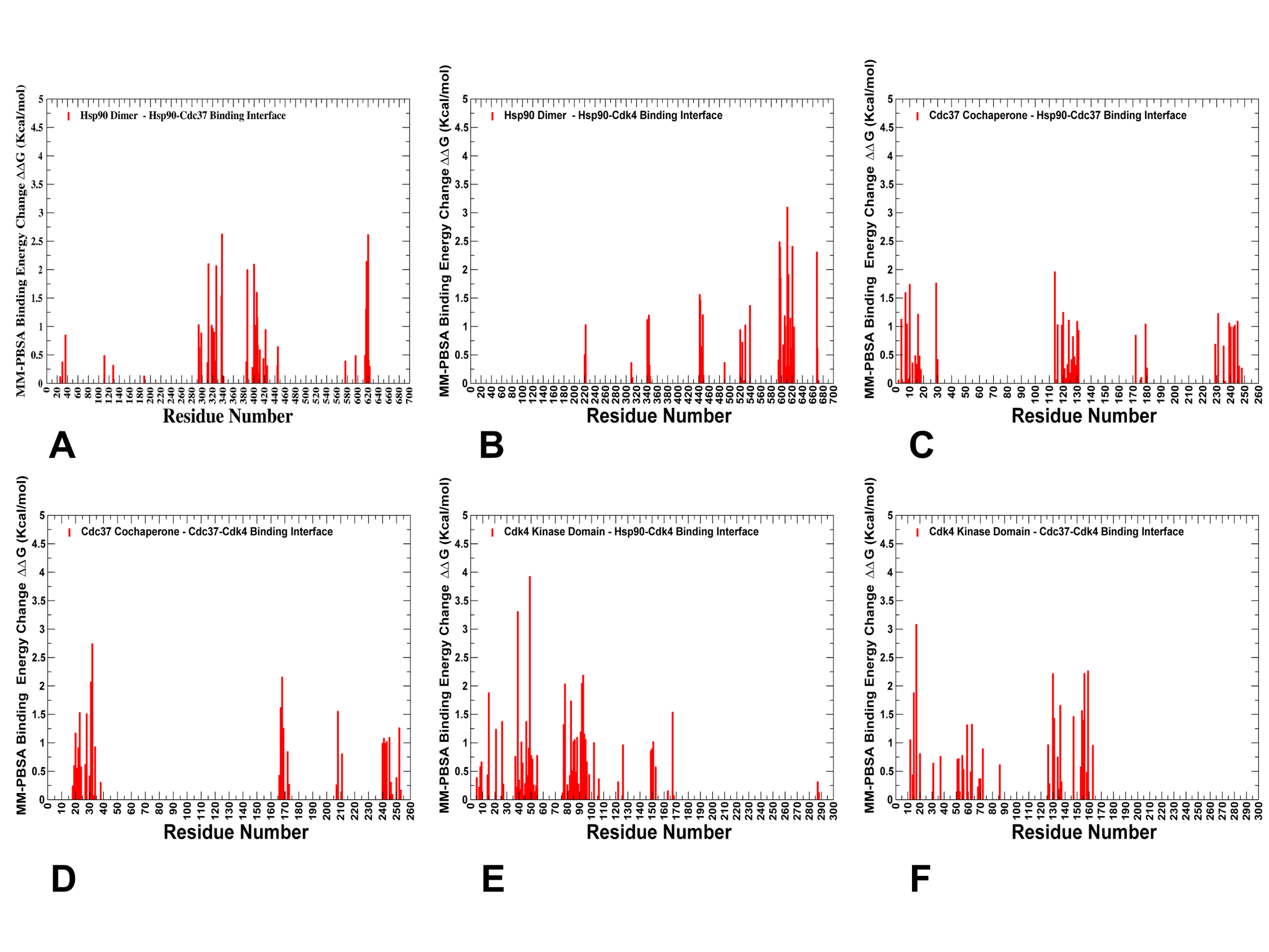

Supplement: S3 Fig — Binding free energy changes obtained through alanine scanning of the interfacial residues in the Hsp90-Cdc37-Cdk4 complex using MM-PBSA approach. Mutation-induced binding free energy changes ΔΔG of the Hsp90 residues involved in the Hsp90-Cdc37 interface (A) and Hsp90-Cdk4 interface (B). Binding free energy changes ΔΔG of the Cdc37 residues forming interfacial contacts with Hsp90 (C) and Cdk4 (D). Binding free energy changes ΔΔG of the Cdk4 residues contributing to the Cdk4-Hsp90 interface (E) and Cdk4-Cdc37 interface (F). If the free energy change between a mutant and the wild type (WT) proteins ΔΔG = ΔG (MT)-ΔG (WT) > 0, the mutation is considered to be destabilizing. The distributions are shown in red-colored bars and highlight only binding interface residues. (TIF) [file pone.0190267.s003.tif]
